# Mitochondrial energy metabolism genes as prognostic biomarkers in clear cell renal cell carcinoma via single-cell and bulk RNA sequencing analyses

**DOI:** 10.1007/s12672-025-04224-1

**Published:** 2025-12-09

**Authors:** Yinqi Peng, Dahao Zhang, Shuangyu Wang, Fu Huang, Haipeng Huang

**Affiliations:** 1 Department of Urology, The Second Affiliated Hospital, Guangxi Medical University, Nanning, Guangxi China; 2 Institute of Transplant Medicine, Guangxi Clinical Research Center for Organ Transplantation, Guangxi Key Laboratory of Organ Donation and Transplantation, The Second Affiliated Hospital of Guangxi Medical University, Nanning, China; 3 Department of Gastroenterology, The Second Affiliated Hospital, Guangxi Medical University, Nanning, Guangxi China

**Keywords:** ccRCC, MMRGs, Single cell analysis, Transcriptome sequencing, Machine learning, Biomarkers

## Abstract

The rising incidence of clear cell renal cell carcinoma (ccRCC) with current treatments offering limited survival benefits and a poor prognosis. Mitochondrial abnormalities impact tumor immunity, progression, and metastasis, and the role of mitochondrial energy metabolism-related genes (MMRGs) in ccRCC remains largely unexplored. This study analyzed TCGA-KIRC, GSE159115, and GSE29609 datasets to identify differentially expressed (DE) MMRGs and their functions. It used LASSO and Cox models to select prognostic MMRGs for model building, created a nomogram (based on independent factors) in TCGA-KIRC (evaluated via calibration and ROC curves), and conducted GSEA, immune cell correlation analyses, TF-miRNA-mRNA network studies, qRT-PCR (ccRCC vs. controls), and WB (RIPA) for biomarker validation. A study of 103 DE-MMRGs highlighted their link to fatty acid metabolism and peroxisome proliferator-activated receptor (PPAR) signaling. Machine learning assessed the prognostic potential of these DE-MMRGs, which yielded a risk model based on six key biomarkers. The constructed prognostic model exhibited outstanding performance in both training and validation sets. This study also explored immune cell relevance and regulatory networks and elucidated complex mitochondrial-tumor interactions. The validation of predictive biomarker expression in clinical samples underscored their role in refining prognostic assessment and therapeutic strategies for ccRCC. In this study, six mitochondrial energy metabolism-related prognosis biomarkers (*COX7B, PPARGC1B, NDUFA11, PFKFB4, NDUFV2, and NDUFA7*) were screened. A risk model was developed to provide a new reference for the prognosis of ccRCC patients.

**Supplementary Information:**

The online version contains supplementary material available at 10.1007/s12672-025-04224-1.

## Introduction

Renal cell carcinoma (RCC) is the most common urological tumor worldwide, with clear cell RCC (ccRCC) being the predominant subtype. It is estimated that over 140,000 people die from RCC each year globally, which imposes a significant burden on society and individuals [54]. While ccRCC can be detected in the early stage and cured with ablative or surgical strategies, one-third of patients will progress to metastasis [32], which threatens the health and even life of the public. It was found that mitochondrial dysfunction is a hallmark of ccRCC and affects metabolic reprogramming and tumor aggressiveness [40]. Critically, ccRCC is recognized as a metabolic disease characterized by profound reprogramming of energy metabolism, including partitioned glycolytic flux, impaired mitochondrial bioenergetics and oxidative phosphorylation (OxPhos), as well as dysregulated lipid metabolism [10, 12, 13, 48]. Notably, ccRCC exhibits one of the highest degrees of immune cell infiltration among solid tumors, characterized by abundant T cells, macrophages, and myeloid-derived suppressor cells (MDSCs) within the tumor microenvironment (TME) [21, 77, 79]. This immunogenic nature directly influences disease progression and response to systemic therapies. Emerging evidence indicates that metabolic reprogramming—including alterations in glucose, fatty acid, and mitochondrial energy metabolism—actively regulates angiogenesis, inflammatory signatures, and immune evasion mechanisms [50, 60].Critically, features of the TME (e.g., cytokine profiles, immune cell composition) heavily modulate therapeutic outcomes for targeted agents and immune checkpoint inhibitors (ICIs) [20, 37–39, 49]. Thus, focusing on the impact of mitochondria in ccRCC not only helps to identify novel prognostic genes but also expands the range of potential therapeutic targets and proposes innovative therapies for the treatment of ccRCC.

Mitochondria, an important catabolic site and a major regulator of cell proliferation and death, provide the majority of bioenergy for organismal functions [74]. Mitochondrial dysfunction has a close relationship with a number of congenital diseases, tumors and inflammation [42, 87]. In ccRCC, mitochondrial dysfunction is a hallmark extending beyond energy metabolism and affects the tumor microenvironment and therapeutic responses [34, 70]. The shift in metabolic pathways, like enhanced glycolysis and tricarboxylic acid (TCA) cycle dysregulation, is a critical aspect of metabolic reprogramming of ccRCC, which is partly orchestrated by mitochondrial dysfunction. The connection between mitochondrial dysfunction and the aggressiveness of ccRCC is complex [15]. Mitochondria also regulate immune responses within the tumor microenvironment. The immune contexture of ccRCC is influenced by the mitochondrial-mediated modulation of immune cell function, including macrophage polarization and cytotoxic T cell activity. This regulation suggests that mitochondrial dysfunction in ccRCC may lead to an immunosuppressive microenvironment. In terms of malignant transformation, the permeability of mitochondrial outer membranes or the transition of mitochondrial permeability is required for early cancer cell survival [9, 29]. Previous research has found that most cancer cells use glycolysis, mitochondrial metabolism and the pentose phosphate pathway to provide necessary resources for fast cell proliferation [82]. Cancer cells, the site for energy metabolism, influence mitochondrial energy processes by altering metabolic patterns and mitochondrial genes. This provides adenosine triphosphate (ATP) for tumors and promotes oncogene-induced mitochondrial reactive oxygen species (ROS) as signaling molecules to modulate cell proliferation[44, 62, 64, 82]. Given the multifaceted role of mitochondria in ccRCC, the potential of mitochondria-targeted therapies is a promising avenue. Therefore, it was speculated that abnormalities in mitochondrial energy metabolism-related genes (MMRGs) may cause aberrations in energy metabolism, which thereby affects ccRCC occurrence and progression. However, the regulatory mechanisms of ccRCC still require further in-depth study.

RCC, particularly ccRCC, continues to challenge clinical management despite the development of targeted therapies and immunotherapies. Although treatments like sunitinib, a tyrosine kinase inhibitor with multiple targets, have demonstrated efficacy in delaying disease progression by inhibiting vascular endothelial growth factor receptors (VEGFRs) and platelet-derived growth factor receptors (PDGFRs), the emergence of drug resistance is a significant clinical hurdle [6]. Similarly, immunotherapies like pembrolizumab and nivolumab, which target programmed death-1 (PD-1) and cytotoxic T-lymphocyte-associated protein 4 (CTLA-4) pathways, have shown promise in attacking cancer cells by unleashing the immune system, but their effectiveness varies among patients [55]. The KEYNOTE-426 trial that compared pembrolizumab plus axitinib with sunitinib monotherapy revealed improved overall survival and objective response rates [68]. Nevertheless, not all patients respond to immunotherapy, which indicates a complex interplay within the immune microenvironment of ccRCC [58]. The limitations of these therapies are manifold, with resistance to targeted therapies potentially arising from the genetic alterations or increased expression of targeted receptors and the heterogeneity of the immune response to immunotherapy [26]. Mitochondria, which play a central role in the metabolic reprogramming of ccRCC, are implicated in drug resistance, but their specific regulatory mechanisms in tumor progression and the potential of mitochondria-targeted therapies remain unclear [67]. For instance, metformin has shown potential in cancer therapy by inhibiting mitochondrial complex I and affects energy production and redox balance, but its clinical application in ccRCC is still exploratory [85]. Comprehensively understanding mitochondrial function in ccRCC and the molecular underpinnings of drug resistance is crucial to developing new therapeutic strategies. Future research should focus on identifying predictive biomarkers for response to therapies and exploring the potential of mitochondria-targeted therapies in combination with existing treatments, which potentially integrates multi-omics data to dissect the intricate relationship between mitochondrial function and drug resistance in ccRCC.

One of the significant challenges in the field is the complexity of mitochondrial energy metabolism and its multifaceted impact on ccRCC [92]. The precise mechanisms by which mitochondrial metabolites exert an influence on tumor behavior and response to therapy are not well understood. In addition, the role of mitochondrial dysfunction in early tumorigenesis and its potential as a treatment target have not been comprehensively explored. This study was aimed at investigating the influence of MMRGs on ccRCC occurrence and progression and uncovering their underlying mechanisms. Are MMRGs expressed abnormally in ccRCC patients? How do MMRGs affect the proliferation and migration of ccRCC cells? Can the regulation of MMRGs provide new strategies for treating ccRCC? In the present study, the prognostic biomarkers related to mitochondrial energy metabolism in ccRCC were identified by bioinformatics based on ccRCC patients' transcriptome data in public databases. Then, immunocorrelation analysis was performed on the biomarkers, and their expression in stromal cell subtypes was analyzed. Related pathways and biological processes enriched in various risk groups were also explored, to provide new ideas for studying the mechanism of ccRCC, the clinical prognosis of tumors, the effect of immunotherapy and the screening of effective antitumor drugs.

## Materials and methods

### Origin of the data

The Cancer Genome Atlas-Kidney Renal Clear Cell Carcinoma (TCGA-KIRC) dataset is currently one of the most comprehensive and authoritative datasets regarding ccRCC. With a vast amount of clinical information and molecular data, it is of great significance for understanding the molecular mechanisms and clinical characteristics of ccRCC. The TCGA-KIRC (ccRCC) dataset was downloaded from the TCGA database (http://cancergenome.nih.gov/) for ccRCC samples, including 542 ccRCC (533 samples with survival information) and 72 normal samples. The data in TCGA-KIRC dataset was subjected to the following preprocessing steps in sequence: (1) by matching with human gene annotation files, only known genes were retained; (2) the aggregate function was used to aggregate multiple probes of the same gene to the maximum value to avoid data bias; (3) the intersect function was used to ensure the sample consistency of the expression matrix with clinical data; (4) normal samples and duplicate samples were excluded.

GSE159115 and GSE29609 datasets were extracted from the Gene Expression Omnibus (GEO) database (http://www.ncbi.nlm.nih.gov/geo/). The GSE159115 dataset (GPL16791) included the single-cell ribonucleic acid (RNA) sequence (scRNA-seq) data of kidney tissue from seven ccRCC samples. The GSE29609 dataset (GPL1708) encompassed the microarray data of frozen kidney tissue from 39 ccRCC samples. The data in GSE29609 dataset underwent the following preprocessing steps in sequence: (1) processing the many-to-many mapping of probes and genes in the data; (2) excluding probes without gene annotation and duplicate genes; (3) row name conversion, gene symbol matching, and human gene screening. GSE159115 and GSE29609 datasets provided additional expression profile data for ccRCC, which complemented the TCGA-KIRC dataset and jointly formed the basis of this research. Their high quality and consistency enabled researchers to more comprehensively analyze the molecular characteristics of ccRCC and validate the findings of this study. The Kyoto Encyclopedia of Genes and Genomes (KEGG) database was accessed by the KEGGREST package in R, and the single-gene enrichment analyses (GSEAs) of low- and high-risk groups were performed by “clusterProfiler” (v 4.4.4) and org.Hs.eg.db (v 3.15.0) packages. MMRGs-related pathways were extracted, and padj < 0.05 was set to filter the results. The 188 MMRGs were extracted from the KEGG pathway (https://www.kegg.jp/kegg/pathway.html) database.

### Identification and functional enrichment of differentially expressed MMRGs

Differentially expressed genes (DEGs) between ccRCC and normal groups were chosen in the TCGA-KIRC dataset by use of the DESeq2 package (v 1.36.1) [46] with |log2FC|> 0.5 and adjusted P value < 0.05. The direct adoption of uncorrected P-value can lead to a high number of false positives. To ensure the reliability of DEGs, an adjusted P-value threshold of < 0.05 is commonly employed to control the false discovery rate, and a |log2FC| threshold of > 0.5 is set to screen for genes with at least a 1.41-fold change in expression levels. This approach ensures both biological significance and minimizes false negatives, which enables the precise identification of truly influential DEGs [78]. The differential analysis was illustrated through the volcano map and heatmap that were plotted via ggplot2 (v 3.4.1) [28] and ComplexHeatmap packages (v 2.12.1) [24], respectively. The ggVennDiagram (v 1.2.2) [17] was used to intersect differential genes and MMRGs, and a Venn diagram was plotted for visualization. DE-MMRGs were filtered by overlapping DEGs and MMRGs. Gene Ontology (GO) and the KEGG enrichment analysis of DE-MMRGs were conducted through the clusterProfiler package (v 4.4.4) [86] (the adjusted P value < 0.05). The R language treemap (v 2.4-3) was utilized for showing the GO function enrichment results. The GOplot (v 1.0.2) of the R language was adopted to generate a Circos plot (commonly referred to as an “Eight Diagrams” plot in some contexts) for visualizing the top 10 KEGG pathways based on functional enrichment analysis. A protein–protein interaction (PPI) network was built based on DE-MMRGs using STRING (https://cn.string-db.org/) (interaction score ≥ 0.7, and other parameters selected website defaults).

### Screening for prognostic biomarkers and risk model construction

A prognostic model was created to investigate whether DE-MMRGs were correlated with the prognosis of ccRCC patients. The univariate Cox algorithm [88] and least absolute shrinkage and selection operator (LASSO) analysis [53] were conducted in the TCGA-KIRC dataset for DE-MMRGs to acquire prognostic biomarkers. A univariate Cox regression analysis was conducted by use of the “survival” package (v 3.3-1) with a p-value threshold of < 0.05. The glmnet package (v 4.1-6) in R was employed, and the parameter family was set to “cox” to implement LASSO logistic regression. The purpose was to select strongly correlated features and obtain a plot of gene coefficients and a plot of the tenfold cross-validation error. The tenfold cross-validation was performed by using the createDataPartition function in the caret package (v 6.0-93) [45] to split the samples in the dataset into a training set and a test set at a ratio of 7:3. To avoid bias caused by random partitioning, the process was run multiple times through a seed loop (from 2235 to 10,000). The optimal model was constructed when the model error was minimized, at which point the lambda value was identified as lambdamin. Patients from the TCGA-KIRC dataset were categorized into low- and high-risk groups following the median value of the risk score computed from biomarkers. The risk score was determined using the formula as follows:

$${\mathrm{Riskscore}}\mathrm{=}{\sum }_{1}^{\mathrm{n}}{\mathrm{coef}}\left({\mathrm{gene}}_{\mathrm{i}}\right)\mathrm{*expression(}{\mathrm{gene}}_{\mathrm{i}}\mathrm{)}$$

The survivalROC package (v 1.0.3) [3] was utilized to calculate the area under the receiver operating characteristic (ROC) curve (AUC) values for ROC curves to assess the predictive accuracy of the model. Kaplan–Meier (KM) survival curves were drawn. In the R package survivalROC, analysis was conducted by inputting variables such as futime (the follow-up time for each subject), fustat (the event status for each subject, where 1 typically signifies the occurrence of an event and 0 indicates no event), riskscore (the risk score assigned to each subject, which is the output of the predictive model) and predict.time (the specific time point at which survival prediction is desired). The plotting process of K-M curves is as follows: conducting survival analysis with the survfit function involved in putting the variable kmfit, which stores the fitted results of the survival object created using the Surv function and risk dataset. To visualize survival curves, the ggsurvplot function was employed, with the variable prog_km capturing the ggplot object representing the survival curve plotted by ggsurvplot. Subsequently, the labs function was called to add a main title and a subtitle to the chart. Furthermore, the customize_labels function was applied again to customize the font styles of the text elements within the prog_km object. Lastly, the risk model was verified with a validation set (GSE29609 dataset).

### Nomogram construction

Independent prognostic analyses were carried out at the TCGA-KIRC dataset, to further investigate the prognosis of clinical-pathological features with risk models. Firstly, a univariate Cox analysis [88] was performed for screening for factors with a P value < 0.05. Then, a proportional hazards (PH) hypothesis test was performed, and factors satisfying the test were included in a multivariate Cox analysis [23] to get independent prognostic factors. The lapply function in R was used, and a hypothesis test was conducted on riskScore, Grade, TNM stage and Stage stage using the cox.zph function. A nomogram was developed based on the unifactorial and multifactorial Cox independent prognostic analysis results by combining the risk score with clinical factors Grade, tumor-lymph node-metastasis (TNM) staging and Stage staging. Variables associated with prognosis were screened by unifactorial screening. The four variables of riskScore, age, Grade, T staging and Stage staging were obtained by PH hypothesis testing for multifactorial Cox analysis. Variables with P value < 0.05 were screened as independent prognostic factors to participate in the construction of the nomogram, and independent prognostic factors were plotted into the nomogram through the R language rms (v 6.2-0) package [61]. Next, the calibration and ROC curves were drawn to judge model performance. ROC curves were plotted using the roc() function from the pROC package for the R language. The x-axis stands for the false positive rate, while the y-axis denotes the true positive rate. The higher the AUC (0.80 < 95% confidence interval (CI) > 0.90) was, the better the performance of the model would be. The ggplot2 package was used to plot a calibration curve. If the curve closely approximated the 45-degree line, it indicated that the predicted probabilities of the model 2.5 Analysis of clinical features.

### Analysis of clinical features

Clinical information was collected by downloading clinical data for TCGA-ccRCC from the TCGA database (http://cancergenome.nih.gov/) to obtain clinicopathological factors such as Grade, TNM stage and Stage stage. Patients with incomplete survival information were excluded. Firstly, the chi-square test was used, and clinical information (age, pathologic-N, gender, pathologic-T, etc.) was analyzed for differences between two risk subtypes in the TCGA-KIRC dataset (N = 533). In addition, comparisons were made between differences in risk scores in different clinical subtypes, and the results were demonstrated via box plots drawn by the ggpubr package (v 0.4.0). Chi-square testing directly assessed whether the differences in the distribution of patient numbers across various clinical subtypes between low- and high-risk groups were statistically significant. If the P-value was below the level of significance (p < 0.05), an association showing statistical significance between categorical variables was considered to exist. That is to say, a difference existed between the two groups.

### Single-gene GSEA

Single-gene GSEA was conducted to find the biological functions and enriched regulatory pathways of two risk subgroups through clusterProfiler (v 4.4.4) [86] and org.Hs.eg.db packages (v 3.15.0) with adjusted P value < 0.05. For analyses using clusterProfiler and org.Hs.eg.db packages, genes were selected for key settings and parameters. “ENTREZID” was chosen as the keyType for gene identifiers. Additionally, “hsa” was selected as the organism, and represented human. Benjamini-Hochberg (“BH”) was chosen as the pAdjustMethod for adjusting P-values. In addition, qvalueCutoff was set to 0.05 as the threshold for filtering significant results. The DESeq2 package was used, and the differential expression analysis of genes was carried out between low- and high-risk groups in the training set. The log2FoldChange values of genes were then sorted, and this sorted list was utilized for GSEA. GSEA was performed based on the KEGG database (perm = 1000), and the results were filtered using a criterion of padj < 0.05. Subsequently, GseaVis (v 0.0.5) was employed for visualizing the top five results (sorted by the normalized enrichment score (NES)) for both low- and high-risk groups separately.

### Immune infiltration and therapeutic analysis

The CIBERSORT algorithm was used for computing the percentages of 22 immune cell subtypes in every sample [7]. The version of CIBERSORT used was CIBERSORTx. Parameters were set to perm = 1000, QN = TRUE, C = 1 i.e., the permutation perm (used to calculate the P-value) and the quantile normalisation QN (set to TRUE in the case of the microarray dataset and FALSE in the case of the sequencing dataset), and the C parameter set the strength of L2 regularization. Then, the Wilcoxon test was used to compare the differences in immune cells between both risk subgroups. Meanwhile, the correlation between differential immune cells was analyzed. The results were demonstrated by the ggiraphExtra tool (v 0.3.0) (https://CRAN.R-project.org/package=ggiraphExtra) plotting radar diagrams. Then, the Spearman method was adopted to analyze the correlation between biomarkers and differential immune cells. Moreover, scatter plots were plotted to demonstrate the associations of risk scores with differential immune cells. The Tumor Immune Dysfunction and Exclusion (TIDE) database (http://tide.dfci.harvard.edu/) was used to obtain TIDE scores for every ccRCC sample [31]. Differences in TIDE scores between differential risk subgroups were compared. The calculation of TIDE scores was based on a set of genes linked to the tumor immune microenvironment. These genes were categorized into two groups: immune-activating (like markers of cytotoxic T cells) and -suppressing genes (like markers of regulatory T cells).

### Drug sensitivity analyses

A total of 138 chemotherapeutic oncology drugs were acquired from the GDSC database (https://www.cancerrxgene.org/) for this study. According to the gene expression data, the half-maximal drug inhibitory concentration (IC50) of every drug was computed for every sample using the pRRophetic package (v 0.5) [18]. The cor.test function, specifically Spearman’s correlation coefficient, was employed for calculating the associations of biomarkers with IC50 values. The results were filtered based on the criteria of P-value < 0.05 and an absolute correlation coefficient (|cor|) ≥ 0.2. This threshold of 0.2 or 0.3 in absolute value is usually used as an indicator of moderate strength correlation in studies, which can be conducive to identifying correlations that may have biological or clinical significance. In the context of gene expression data, where changes in expression can be relatively subtle, a correlation of 0.2 may already be considered significant. To visualize the filtered correlation results, the ggplot2 package was used to create a bar chart. This visualization aided in understanding which chemotherapeutic drugs exhibited a potentially meaningful correlation with risk scores. Next, the difference in the IC50 of each drug with a risk score was computed.

### Construction of regulatory networks

The multiMiR package (v 1.18.0) was employed to predict micro RNAs (miRNAs) targeting biomarkers based on miRanda, miRDB and miCrocosm databases. Predictions from the three databases were crossed. Next, the NetworkAnalyst database (https://www.networkanalyst.ca/) was utilized for the prediction of transcription factors (TFs) targeting biomarkers. In addition, miRNAs regulate gene expression via base complementary pairing with the 3'UTR of mRNAs through their seed regions, which typically span two to eight nucleotides. They often identify potential target genes based on the complementarity of these seed regions. Prediction tools normally identify potential target genes in light of the complementarity of these seed regions. Furthermore, miRNA-messenger RNA (mRNA) binding energy also affects the prediction results, with lower binding free energy indicating stronger interactions. A t-test was used for assessing the associations of miRNAs with their targets, and only genes with statistically significant correlations were selected. Genes involved in model construction were pasted into the gene list input box and appropriate species were selected. The “Network” option was selected to construct the co-expression network. After that, the transcription factor (TF)-miRNAs-mRNA regulatory network was obtained by Cytoscape [76]. In addition, the co-expression networks of biomarkers were explored in this study using GeneMANIA (http://genemania.org/). Each node represents a gene, and edges represent interactions between genes. Cytoscape was used for visualization.

### Single-cell RNA-seq analysis

The GSE159115 dataset was downloaded from the GEO database to obtain single-cell sequencing data from 7 KIRC (ccRCC) tumor tissues. Then the CreateSeuratObject function of the Seurat package (v 4.0.5) was used to filter the single-cell sequencing data [84]. Firstly, exclude genes expressed in fewer than three cells; second, retain cells where nCount_RNA falls between 200 and 90,000, nFeature_RNA between 200 and 9,000, and mitochondrial gene proportion remains below 10%; Concurrently, to exclude interference from doublet cells in the analysis, the scDblFinder package (v 1.14.0) was employed to identify and remove doublet cells from the remaining population [19]. Next, NormalizeData was used for the normalization of the data. Subsequently, the “vst” method and the FindVariableFeatures function were used for identifying 2,000 genes whose expression differed significantly from cell to cell for subsequent analysis. The normalised data underwent principal component analysis (PCA), with the top 30 principal components selected for subsequent analysis. Batch effects were corrected using the RunHarmony() function from the Harmony package (v 1.2.3) [36]. Immediately afterwards, the FindNeighbors and FindClusters functions from the Seurat package (v 4.0.5) were used for performing an unsupervised cluster analysis [69], and Uniform Manifold Approximation and Projection (UMAP) was used to visualize the clusters. The unsupervised cluster analysis was conducted based on the PCA dimensionality reduction results by using FindNeighbors and FindClusters functions. The parameter nfeatures = 2,000 was set and the method was selected as “vst”. In the meantime, the parameter resolution = 0.5 was set.

FindAllMarkers was applied to find marker genes for each cluster. Marker genes for each cluster were compared with those for each cell type in the CellMarker database to determine the subpopulation type of cells. The SingleR algorithm [1] was utilized to assist in the validation of the identified cell types. The algorithm automatically annotated single cells by comparing the expression profiles of single cells in the test dataset with those of a reference dataset of cell types. Finally, the expression of biomarkers was detected in each cell subtype, and violin and dot plots were used for the vitalization of the results. were accurate.

### Sample selection

Five ccRCC tissue samples and five adjacent normal tissue samples were collected from patients undergoing surgical resection at The Second Affiliated Hospital of Guangxi Medical University. Informed consent was obtained from all patients, and the research protocol received approval from the Ethical review of clinical research projects of the Medical Ethics Committee of the Second Affiliated Hospital of GuangXi Medical University (Approval No. 2024-KY-0485). The samples were immediately frozen in liquid nitrogen after resection and stored at − 80 °C until further analysis. Clinical data, including patient age, gender, tumor stage, and histopathological details, were also collected to ensure comprehensive analysis.

### Ethics approval and consent to participate

The study was conducted in accordance with the Declaration of Helsinki. The study utilizing five paired surgical tissues (five ccRCC and five adjacent normal tissues) collected from patients undergoing surgical resection at The Second Affiliated Hospital of Guangxi Medical University was approved by the Medical Ethics Committee of The Second Affiliated Hospital of Guangxi Medical University (Approval No. 2024-KY-0485). Sample source: Residual tissues from patients undergoing nephrectomy at The Second Affiliated Hospital of Guangxi Medical University. Enrollment type: Prospective collection of clinical samples with written informed consent. Study period: 2024-12-01 to 2025-01-05. Informed consent: Written informed consent was obtained from all participants, witnessed and formally documented.

### Quantitative reverse transcription polymerase chain reaction

Subsequently 50 mg of each was taken to extract sample RNA using TRIzol reagent (Ambion, the United States of America (USA)). Then, 1 ul of RNA was extracted for concentration using a NanoPhotometer N50, and its purity and concentration were noted to calculate the uptake volume for reverse transcription operations, which were performed by use of the SureScript-First-strand-cDNA-synthesis-kit (servicebio, Wuhan, China) as per the instructions of the manufacturer. Briefly, the instructions included 25, 50, 85 and 4 °C for 5 min, 15 min, 5 s and storage, respectively. The CFX96 real-time quantitative fluorescence polymerase chain reaction (PCR) machine was utilized to conduct quantitative reverse transcription-PCR (qRT-PCR) on the reverse transcription product complementary deoxyribonucleic acid (cDNA). The qRT-PCR was completed strictly following the instructions of the manufacturer for 2xUniversal Blue SYBR Green qPCR Master Mix (Servicebio, Wuhan, China). The qPCR reaction mixture comprised 3 µL of cDNA, 5 µL of 2xUniversal Blue SYBR Green qPCR Master Mix (Servicebio, Wuhan, China), 1 µL of forward primer (Tsingke, China) at a concentration of 10 µM, and 1 µL of reverse primer (Tsingke, China) also at a concentration of 10 µM. In addition, the CFX96 Real-Time PCR Detection System (Bio-Rad, China) was used to perform qPCR according to the following steps: 1-min pre-denaturation at 95 ˚C, 40 cycles of denaturation at 95 ˚C for 20 s, 20-s annealing at 55 ˚C and 30-s extension at 72 ˚C. The 2^−ΔΔCT^ method was applied for normalizing gene expression to glyceraldehyde-3-phosphate dehydrogenase (GAPDH). Primer sequence information is shown in Table 1.

**Table 1:** Primer sequences for qRT-PCR

Primer	Sequence
COX7B F	GCCACCAGAAACGTACACCT
COX7B R	CCTTTGGGGTAACTCTGCCA
PPARGC1B F	TCAACTATCTCGCTGACACGC
PPARGC1B R	TCTGGAAGAGCTCGGAGTCA
NDUFA11 F	AGCACCACCAGTATTGCCAG
NDUFA11 R	CAATCCCGTAGTTGTGCGTG
PFKFB4 F	GACCAACTGCCCAACTCTCA
PFKFB4 R	CAAGTCTGGGCGCTGATGTA
NDUFV2 F	TGGGGAAGGTGAACAGTGTG
NDUFV2 R	CCCATTCTGCCTTTGGGCTA
NDUFA7 F	GAATCTGTGCCCCCTTCCAT
NDUFA7 R	GTCACCGCCTTCTTCTCAGT
GAPDH F	CGAAGGTGGAGTCAACGGATTT
GAPDH R	ATGGGTGGAATCATATTGGAAC

### Western blot analysis

Briefly, tissue samples were homogenized in RIPA buffer supplemented with protease and phosphatase inhibitors. The lysates were centrifuged at 14,000*g* for 15 min at 4 °C, and the supernatants were collected. Protein concentrations were determined using the Bradford assay (Bio-Rad). Equal amounts of protein (30 μg) from each sample were resolved by sodium dodecyl sulfate–polyacrylamide gel electrophoresis (SDS-PAGE) and transferred onto polyvinylidene fluoride (PVDF) membranes. Membranes were blocked with 5% non-fat dry milk in Tris-buffered saline containing 0.1% Tween-20 (TBST) for 1 h at room temperature. Primary antibodies were diluted in blocking buffer and incubated overnight at 4 °C with gentle shaking. The following primary antibodies were used: anti-COX7B (abcam, ab140629), anti-NDUFA7 (abcam, ab140871), anti-NDUFA11 (abcam, ab183707), anti-NDUFV2 (abcam, ab183715), anti-PFKFB4 (abcam, ab137785), and anti-PPARGC1B (thermo fisher scientific, MA5-32,765). After washing with TBST, membranes were incubated with horseradish peroxidase (HRP)-conjugated secondary antibodies for 1 h at room temperature. Immunoreactive bands were visualized using enhanced chemiluminescence (ECL) substrate (Thermo Fisher Scientific) and detected using a chemiluminescence imaging system (Bio-Rad). Band intensities were quantified using ImageJ software (NIH) and normalized to GAPDH as an internal control to ensure equal protein loading and transfer efficiency.

### Statistical analysis

The R language (v 4.3.1) was employed to carry out all bioinformatics analyses. Errors, missing values or outliers in data were removed or corrected using R. Missing data were identified and processed, including the interpolation or deletion of missing value records. Multiple units of measure in clinical data were converted to standard units. The naming of categorical variables was standardized. The scale of the data was adjusted to make it suitable for subsequent analyses. The Wilcoxon test was applied for comparing the data of different groups (P < 0.05).

## Results

### DE-MMRGs in ccRCC

**Fig.1:**
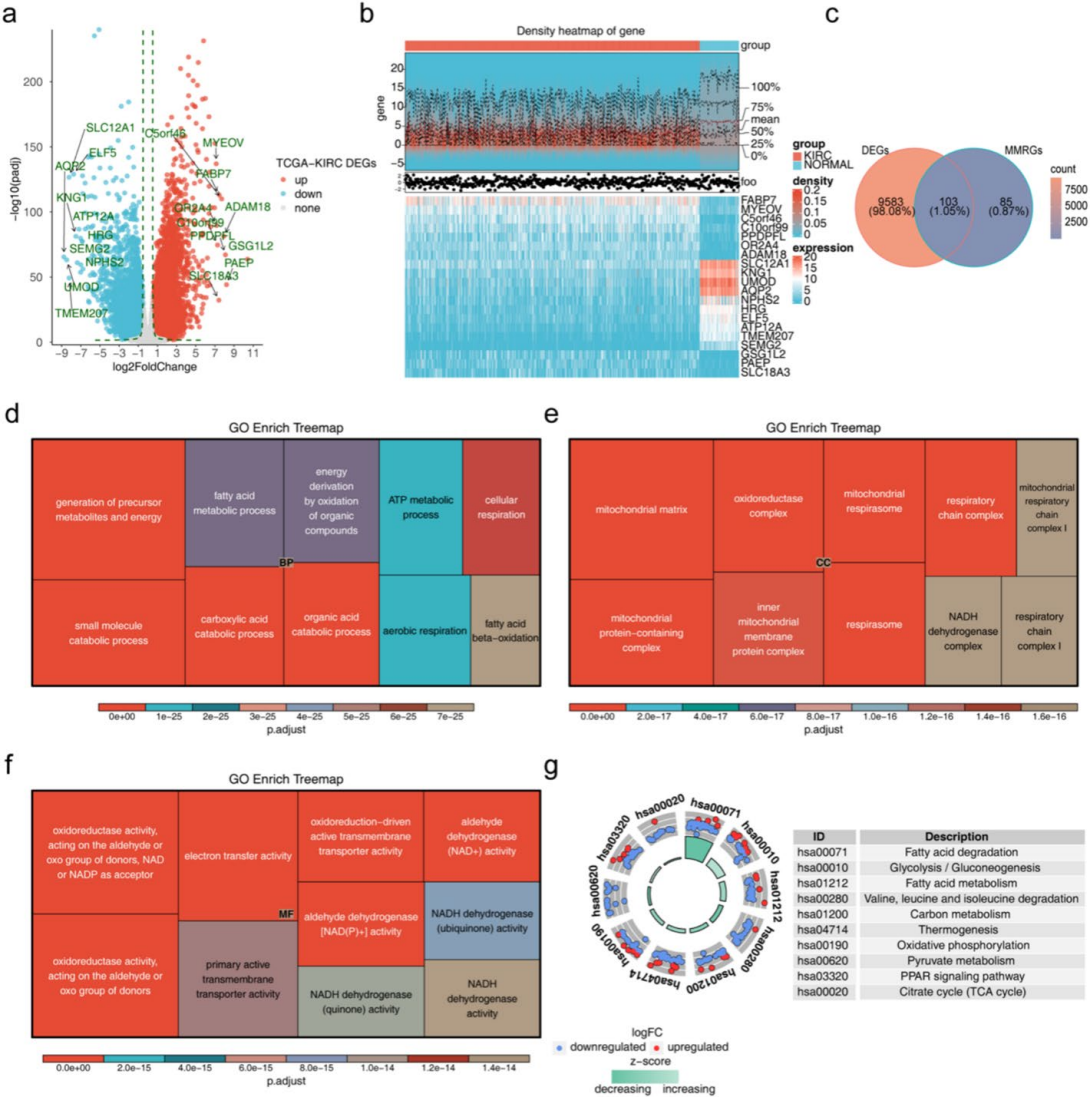
DEGs obtained in ccRCC and normal group. **a **Volcano plot of DEGs. Red, blue and gray dots represent up-regulated genes, down-regulated genes, and genes without significant differences or with small fold changes, respectively. **b** Heatmap of differential gene analysis. The upper half displays a heatmap of expression density, while the lower half shows the expression heatmap. The expression heatmap showed DEGs that up- and down-regulate the top 10 in KIRC and normal samples. **c** Venn diagram of DEGs and MMRGs. **d** Enrichment treemap of GO-BP of DE-MMRGs. **e** Enrichment treemap of GO-CC of DE-MMRGs. **f** Enrichment treemap of GO-MF of DE-MMRGs. (g) KEGG pathway enrichment of DE-MMRGs

A total of 9,686 DEGs (6,152 up- and 3,534 down-regulated DEGs) between ccRCC and normal groups were acquired (Fig. 1a). The top 10 up- and down-regulated DEGs are displayed in Fig. 1b. The top up-regulated DEGs included fatty acid binding protein 7 (FABP7), myeloma-overexpressed gene protein (MYEOV), chromosome 5 open reading frame 46 (C5orf46). The top down-regulated DEGs contained solute carrier family 12 (sodium/potassium/chloride transporters), member 1 (SLC12A1), Kininogen 1 (KNG1), Uromodulin (UMOD). They were chosen because they were most statistically significant. That is, the expression changes of these genes were the least likely to be caused by random errors, and therefore more likely to have real biological significance. Next, to investigate mitochondrial energy metabolism-related genes, 103 differentially expressed mitochondrial metabolism-related genes (DE-MMRGs) were identified through RNA sequencing and bioinformatic analysis Genes such as hydroxyacyl-coenzyme A dehydrogenase (HADH), PFKFB4, ATP12A, acyl-CoA dehydrogenase short/branched chain (ACADSB), phosphofructokinase, platelet (PFKP), acetyl-CoA acyltransferase 1 (ACAA1), ADP-dependent glucokinase (ADPGK), acyl coenzyme A synthetase long chain family, member 4 (ACSL4), pyruvate dehydrogenase beta (PDHB), etc.) were filtered by overlapping DEGs (Fig. 1c). In addition, enrichment analysis demonstrated that DE-MMRGs involved 434 GO entries (313 biological processes (BPs), 29 cellular components (CCs) and 92 molecular functions (MFs)) and 53 KEGG pathways. These DE-MMRGs were primarily enriched to GO- BP entries like small molecule catabolic process and fatty acid metabolic process, GO-CC entries like mitochondrial matrix and oxidoreductase complex, and GO-MF entries like electron transfer activity and NADH dehydrogenase activity (Fig. 1d–f). Pyruvate metabolism, peroxisome proliferator-activated receptor (PPAR) signaling pathway, etc. were enriched in KEGG pathways (Fig. 1g). To observe the association between DE-MMRGs, a PPI network was constructed (Fig. S1). Among DE-MMRGs, proteins such as ACAA1, enoyl-CoA, hydratase/3-hydroxyacyl CoA dehydrogenase (EHHADH) and acyl-coenzyme A oxidase 1 (ACOX1) showed stronger associations with other proteins.

### *COX7B, PPARGC1B, NDUFA11, PFKFB4, NDUFV2 *and *NDUFA7* defined as prognostic biomarkers and modeled for a favorable prognosis

**Fig. 2:**
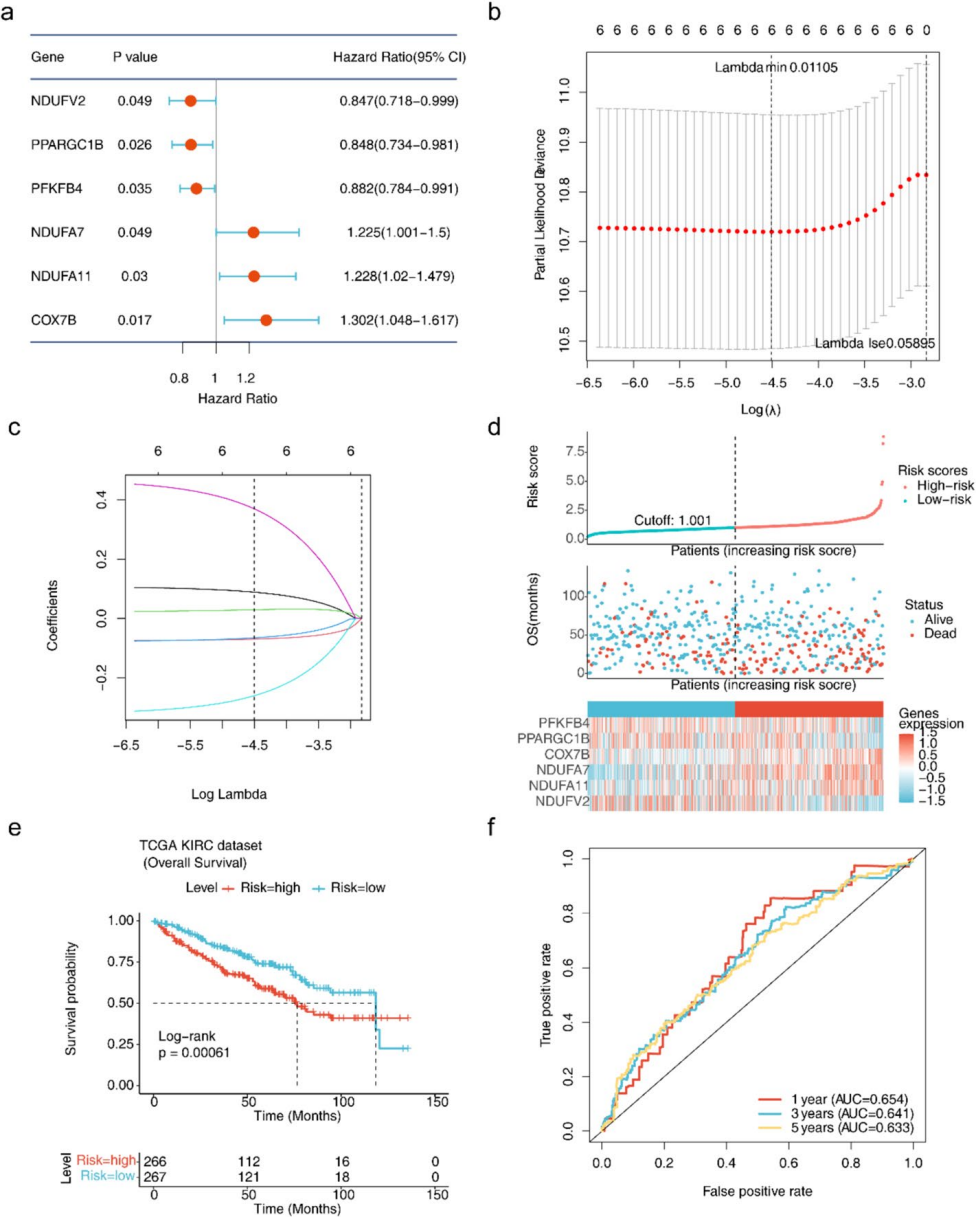
Biomarker screening and prognostic model construction and evaluation. **a** Forest plot of univariate Cox regression analysis. **b**, **c** LASSO regression analysis. **d-1** Risk curves for the low- and high-risk subgroups of the training set. Red represents the high-risk group and blue stands for the low-risk group. **d-2** Scatterplots of OS for the low- and high-risk subgroups of the training set. Red means that the patient is dead and blue means that the patient is alive. **d-3** Heatmaps of the expression of prognostic biomarkers for the low- and high-risk subgroups of the training set. **e** Survival curves for the low- and high-risk subgroups of the training set. **f** ROC curves for the 1/3/5 years of the training set

**Fig. 3:**
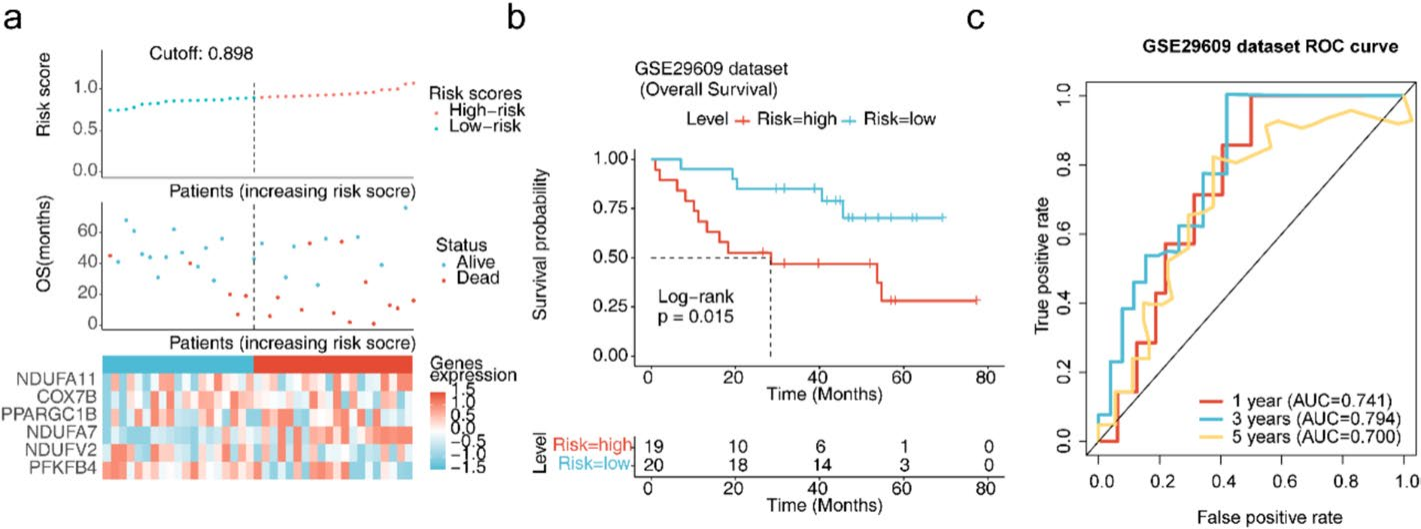
Validation of risk models. **a-1** Risk curves for low- and high-risk subgroups for GSE29609. Red represents the high-risk group and blue stands for the low-risk group. **a-2** Scatterplots of OS for low- and high-risk subgroups for GSE29609. Red means that the patient is dead and blue means that the patient is alive. **a-3** Heatmaps of the expression of prognostic biomarkers in low- and high-risk subgroups. Red represents high expression and blue stands for low expression. **b** Survival curves for low- and high-risk subgroups. **c** ROC curves for 1/3/5-year survival

Based on the DE-MMRGs, prognostic biomarkers were further obtained. In total, six biomarkers (*COX7B, PPARGC1B, NDUFA11, PFKFB4, NDUFV2 and NDUFA7*) were acquired by the univariate Cox algorithm and LASSO analyses (Fig. 2a–c). Among these biomarkers, NDUFV2 (hazard ratio (HR) = 0.847, 0.718 < 95% CI > 0.999), PPARGC1B (HR = 0.848, 0.734 < 95% CI > 0.981) and PFKFB4 (HR = 0.882, 0.784 < 95% CI > 0.991) were protective factors (HR < 1). In addition, NDUFA7 (HR = 1.225, 1.001 < 95% CI > 1.5), NDUFA11 (HR = 1.228, 1.02 < 95% CI > 1.479) and COX7B (HR = 1.302, 1.048 < 95% CI > 1.617) were risk factors (HR > 1) (Fig. 2a). Then, patients were separated into groups with high (n = 266) and low risks (n = 267) based on the median value (Fig. 2d). Survival analysis curves demonstrated that the high-risk group of the TCGA-KIRC dataset reported a lower survival rate (Fig. 2e). ROC curves suggested that the model showed decent predictive performance with AUCs above 0.61 (1-, 3- and 5-years) (Fig. 2f). After that, the GSE29609 dataset was utilized for evaluating predictive model performance. Patients in GSE29609 were classified into high- and low-risk groups based on the median risk score (Median Riskscore = 0.898). As displayed by the survival curves and risk profile plots of the external validation set, the results were in line with the TCGA-KIRC dataset (Fig. 3a, b). The AUC values for the GSE29609 dataset were all greater than 0.70 (0.80 < 95% CI > 0.90), which indicated the better predictive performance of the model **(**Fig. 3c).

### High predictive value of the nomogram determined by risk score and age for ccRCC

**Fig. 4:**
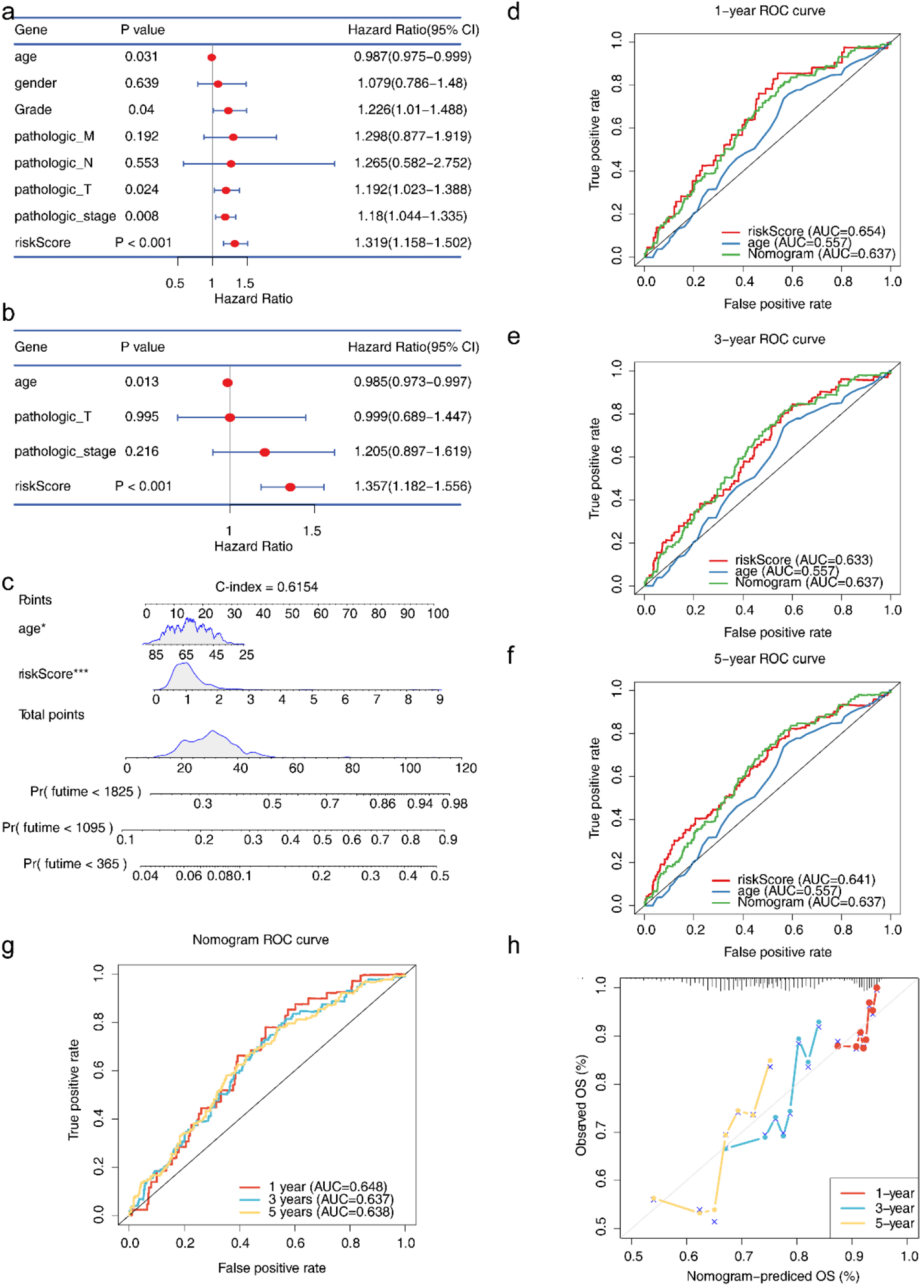
Analysis of risk scores and clinical characteristics

PH hypothesis testing found that Grade staging did not fulfill the PH hypothesis, whereas riskScore, TNM staging and Stage staging did. The independent prognostic analysis results indicated that two significant factors (risk score and age) were acquired (Fig. 4a, b). The nomogram according to prognostic factors was used for predicting the overall survival (OS) of patients (1-, 3- and 5-years) (C-index = 0.6154) (Fig. 4c). AUC values exceeded 0.63, which suggested that the nomogram model was a better predictor than the risk score alone (Fig. 4d–g).The calibration curve illustrated that the nomogram had a decent predictive accuracy for patient survival at 1-, 3-, and 5-years (Fig. 4h).

### Analysis of risk scores and clinical characteristics

**Fig. 5:**
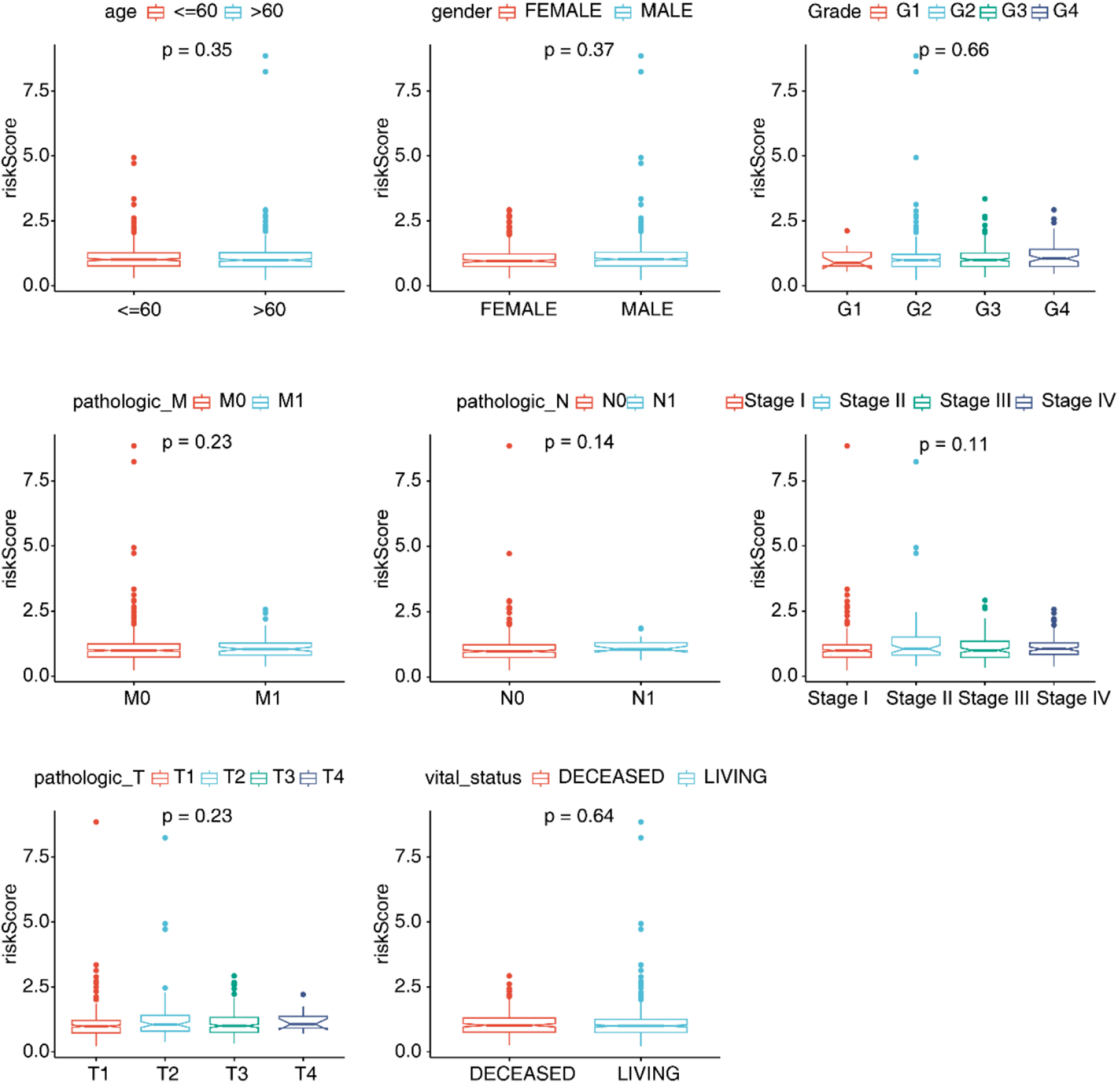
Boxplots of risk scores in different clinical subtypes

After independent prognostic factors were acquired, clinical characteristics were further analyzed. Analysis of variance manifested that the number of patients in both risk subgroups showed significant differences in vital-status, OS and pathologic-M (P value < 0.05) (Table 2). Meanwhile, no significant differences were observed in the risk scores of subgroups with different clinical characteristics, which suggested that risk scores were independent (Fig. 5).

**Table 2:** Results of clinical characterisation

	**Total**	**Risk**		***Pvalue***
		**High**	**Low**	
*Age (year)*				
Mean (SD)	60.6 (± 12.1)	59.9 (± 11.6)	61.4 (± 12.7)	0.19
*Gender*				
Female	188 (35.3%)	83 (31.2%)	105 (39.3%)	0.057
Male	345 (64.7%)	183 (68.8%)	162 (60.7%)	
*Vital_status*				
Alive	358 (67.2%)	161 (60.5%)	197 (73.8%)	0.001
Dead	175 (32.8%)	105 (39.5%)	70 (26.2%)	
*OS (Months)*				
Mean (SD)	47.2 (± 30.4)	44.5 (± 30.9)	50.0 (± 29.7)	0.027
*Grade*				
G1	14 (2.7%)	6 (2.3%)	8 (3.0%)	0.69
G2	229 (43.6%)	110 (42.3%)	119 (44.9%)	
G3	206 (39.2%)	102 (39.2%)	104 (39.2%)	
G4	76 (14.5%)	42 (16.2%)	34 (12.8%)	
*pathologic_M*				
M0	422 (84.2%)	202 (82.4%)	220 (85.9%)	0.33
M1	79 (15.8%)	43 (17.6%)	36 (14.1%)	
*pathologic_N*				
N0	240 (93.8%)	114 (89.8%)	126 (97.7%)	0.01
N1	16 (6.2%)	13 (10.2%)	3 (2.3%)	
*pathologic_T*				
T1	273 (51.2%)	132 (49.6%)	141 (52.8%)	0.51
T2	69 (12.9%)	39 (14.7%)	30 (11.2%)	
T3	180 (33.8%)	88 (33.1%)	92 (34.5%)	
T4	11 (2.1%)	7 (2.6%)	4 (1.5%)	
*pathologic_stage*				
Stage I	267 (50.3%)	127 (47.7%)	140 (52.8%)	0.34
Stage II	57 (10.7%)	32 (12.0%)	25 (9.4%)	
Stage III	123 (23.2%)	59 (22.2%)	64 (24.2%)	
Stage IV	84 (15.8%)	48 (18.0%)	36 (13.6%)

### Enrichment of two risk groups to separate pathways

**Fig. 6:**
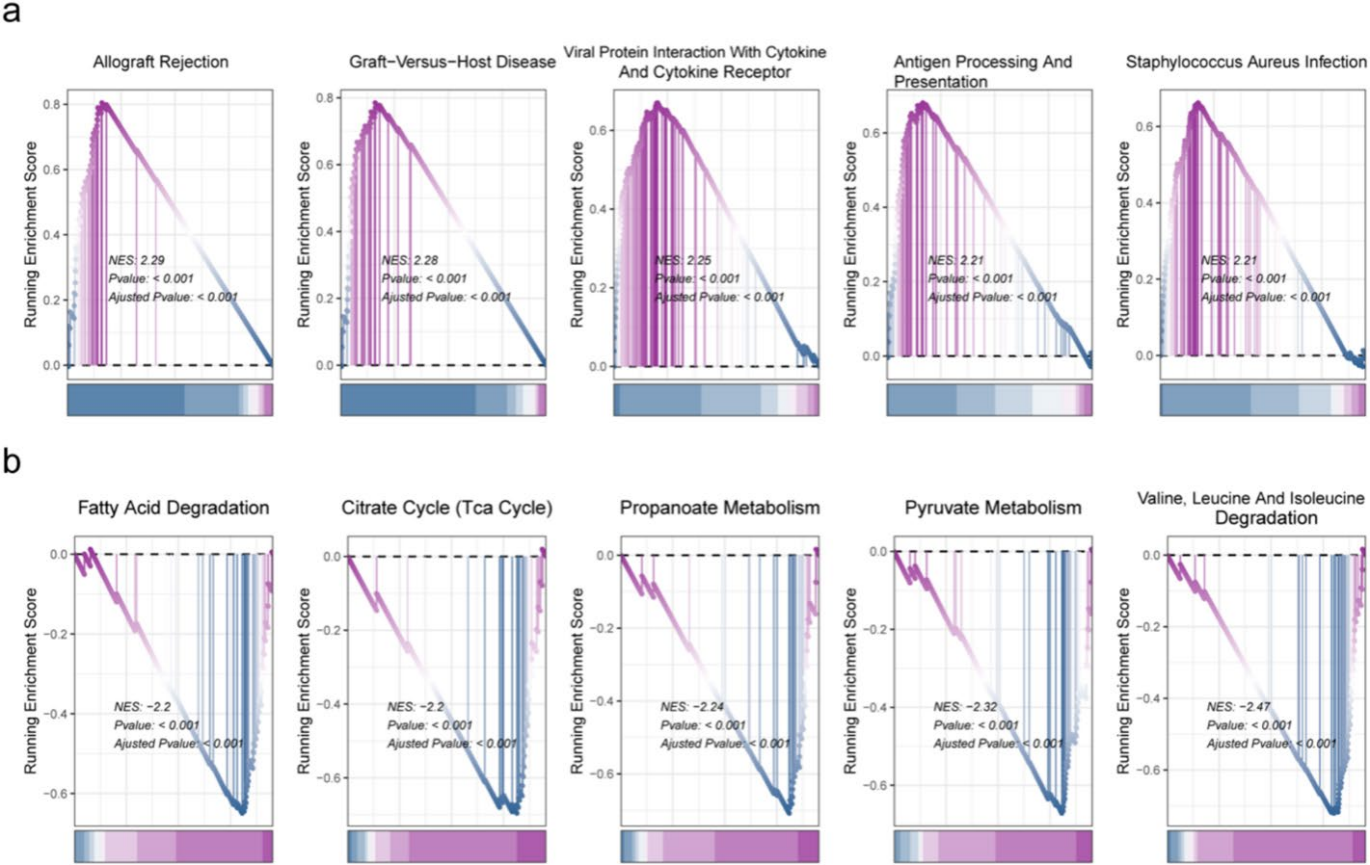
Analysis of functional differences between risk subgroups. **a **Ridge diagram of the top five pathways in the high-risk group. **b** Ridge diagram of the top five pathways in the low-risk group

The KEGG pathway enrichment analysis of the two risk subgroups showed that pathways such as allograft rejection, antigen processing, cytokine receptor and presentation were chiefly enriched in the high-risk group (Fig. 6a). In addition, citrate cycle (TCA cycle), pyruvate metabolism, propanoate metabolism, fatty acid degradation, etc. were mainly enriched in the low-risk group (Fig. 6b).

### Possible opposing roles of *NDUFA11* and *PPARGC1B* in light of the role of regulatory T cells in ccRCC

**Fig. 7:**
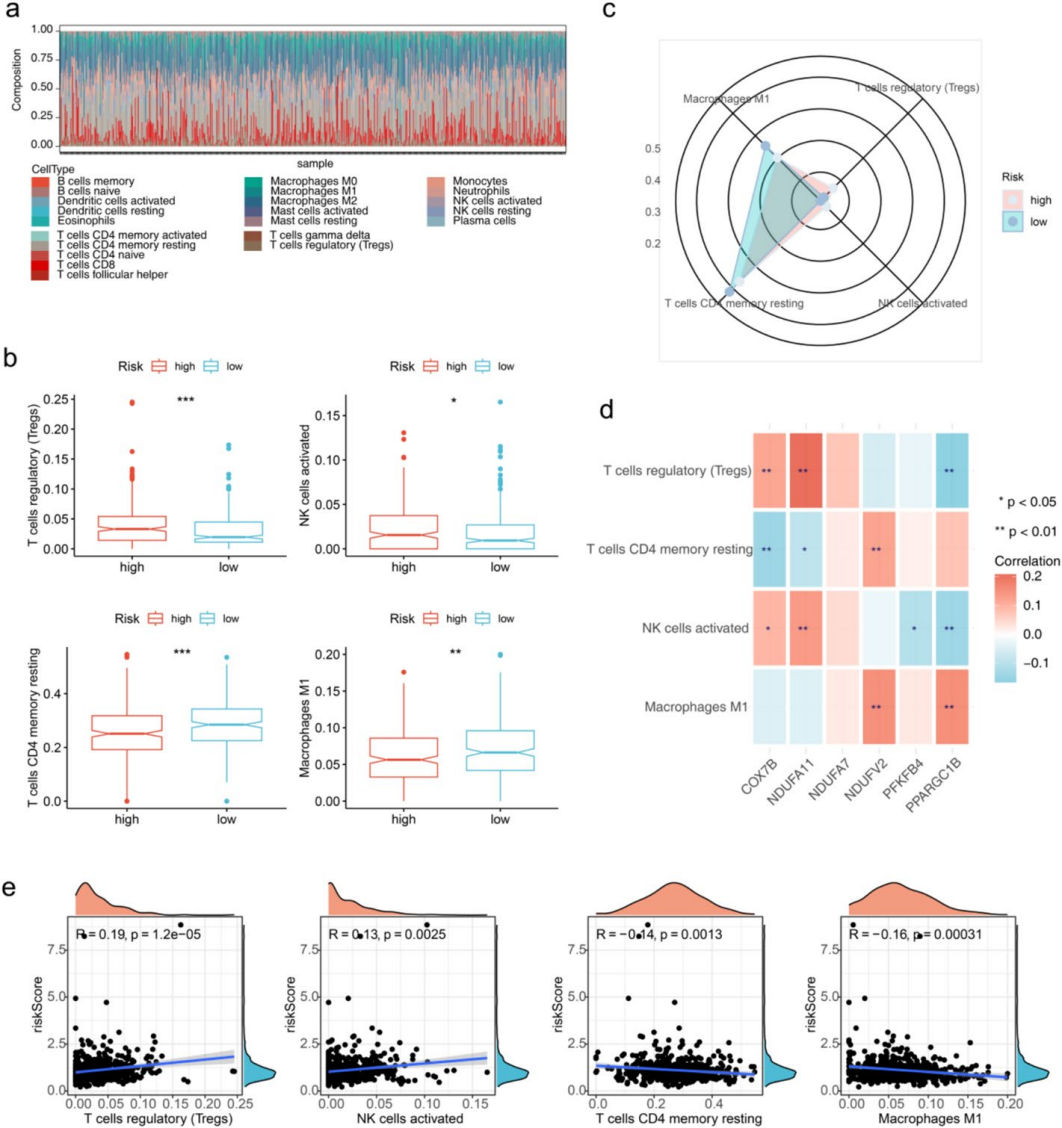
Immune-related analyses of biomarkers and risk scores. **a **Histogram of the distribution of immune cells in the TCGA-KIRC dataset. **b** Box plots of the proportions of differentiated immune cells in low- and high-risk groups. *p < 0.05, **p < 0.01 and ***p < 0.001. **c** Radar chart of differential immune cell correlations. **d** Heatmap of prognostic biomarkers correlating with differential immune cells. Blue represents a negative correlation and red stands for a positive correlation. **e** Scatterplot of risk score and differential immune cell correlation

Increasing evidence has elucidated the significance of the immune microenvironment in predicting the prognostic outcome of tumors. In this study, four types of immune cells differed significantly between risk subgroups (Fig. 7a–c). Among them, the percentages of resting memory clusters of differentiation 4 (CD4) T and activated natural killer (NK) cells were greater in the high-risk group. Correlation analysis showed that regulatory T cells (Tregs) had a stronger negative correlation with M1 macrophages and resting memory CD4 T cells (Fig. 7c). The correlation analysis of prognostic biomarkers with differential immune cells illustrated that Tregs had the strongest positive association (r = 0.207585464) with NDUFA11 and the strongest negative relationship (r = −0.166033293) with PPARGC1B (Fig. 7d). Activated NK cells (r = 0.13, P = 0.0025) and Tregs (r = 0.19, P = 1.2e–05) were positively associated with a risk score while resting memory CD4 T cells (r = −0.14, P = 0.0013) and M1 macrophages (r = −0.16, P = 0.00031) showed a negative correlation with it (Fig. 7e).

### Possible important roles of salubrinal and metformin in ccRCC

**Fig. 8:**
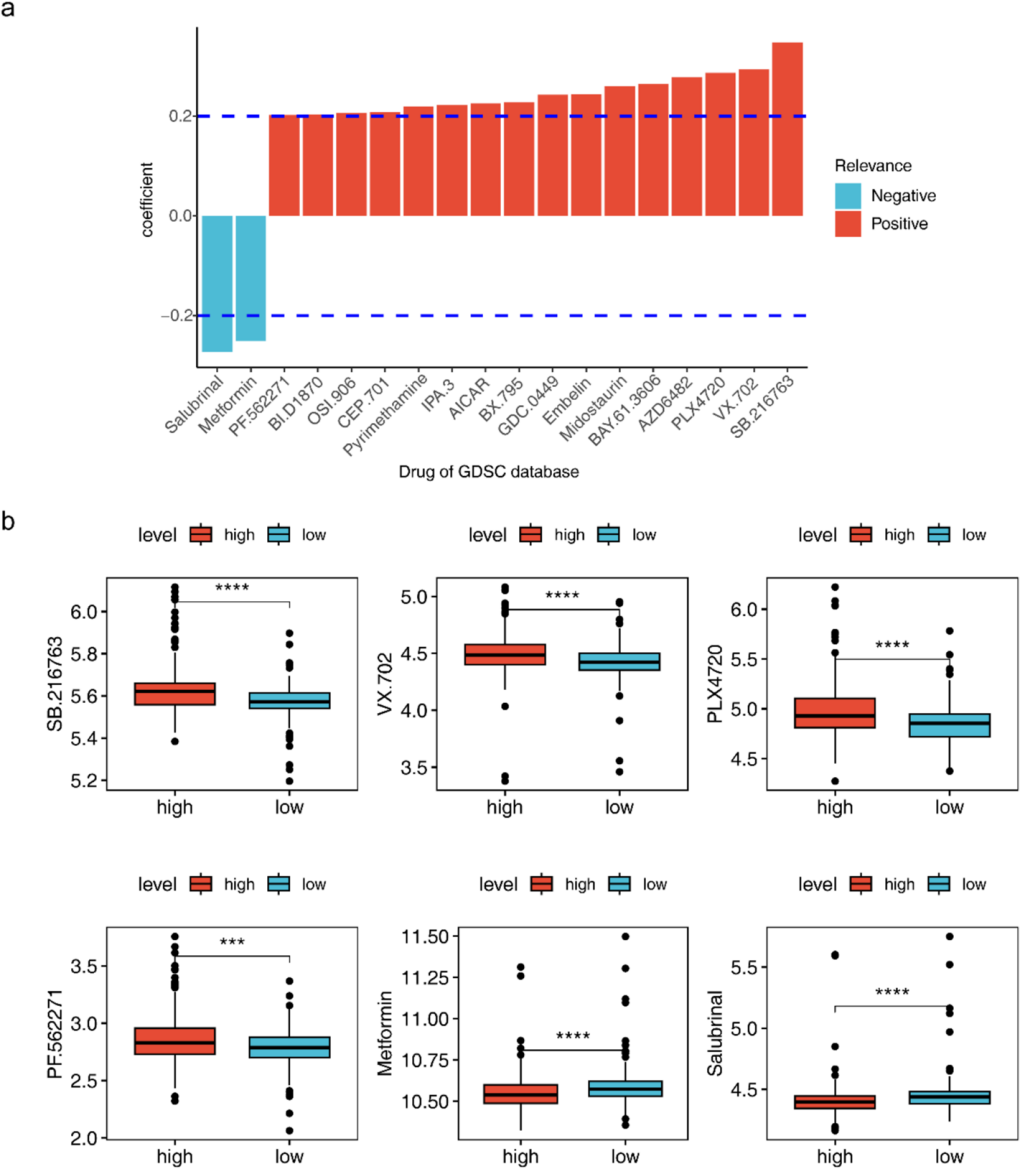
Drug sensitivity analyses between differential risk subgroups. **a **Bar chart of risk scores and drug correlations. **b** Box plots of IC50 for drugs in low- and high-risk groups

The association analysis demonstrated that risk score was negatively related to two drugs (Salubrinal and Metformin) and positively associated with 16 drugs (PLX4720, VX.702, SB.216763, etc.) (Fig. 8a). The IC50 values of these 18 drugs were analyzed for the differences between low- and high-risk groups, which were significant for all 18 drugs (p < 0.001), and visualized using box-and-line plots for the positive and negative correlations of the top three drugs (PF.562271, Metformin, Salubrinal, SB.216763, VX.702, PLX4720). The high-risk group had lower IC50 values of Metformin and Salubrinal, while the low-risk one had greater IC50 values of PF.562271, SB.216763, VX.702 and PLX4720, which was in agreement with the correlation analysis results (Fig. 8b).

### TF-miRNA-mRNA regulatory and co-expression networks of prognostic biomarkers

**Fig. 9:**
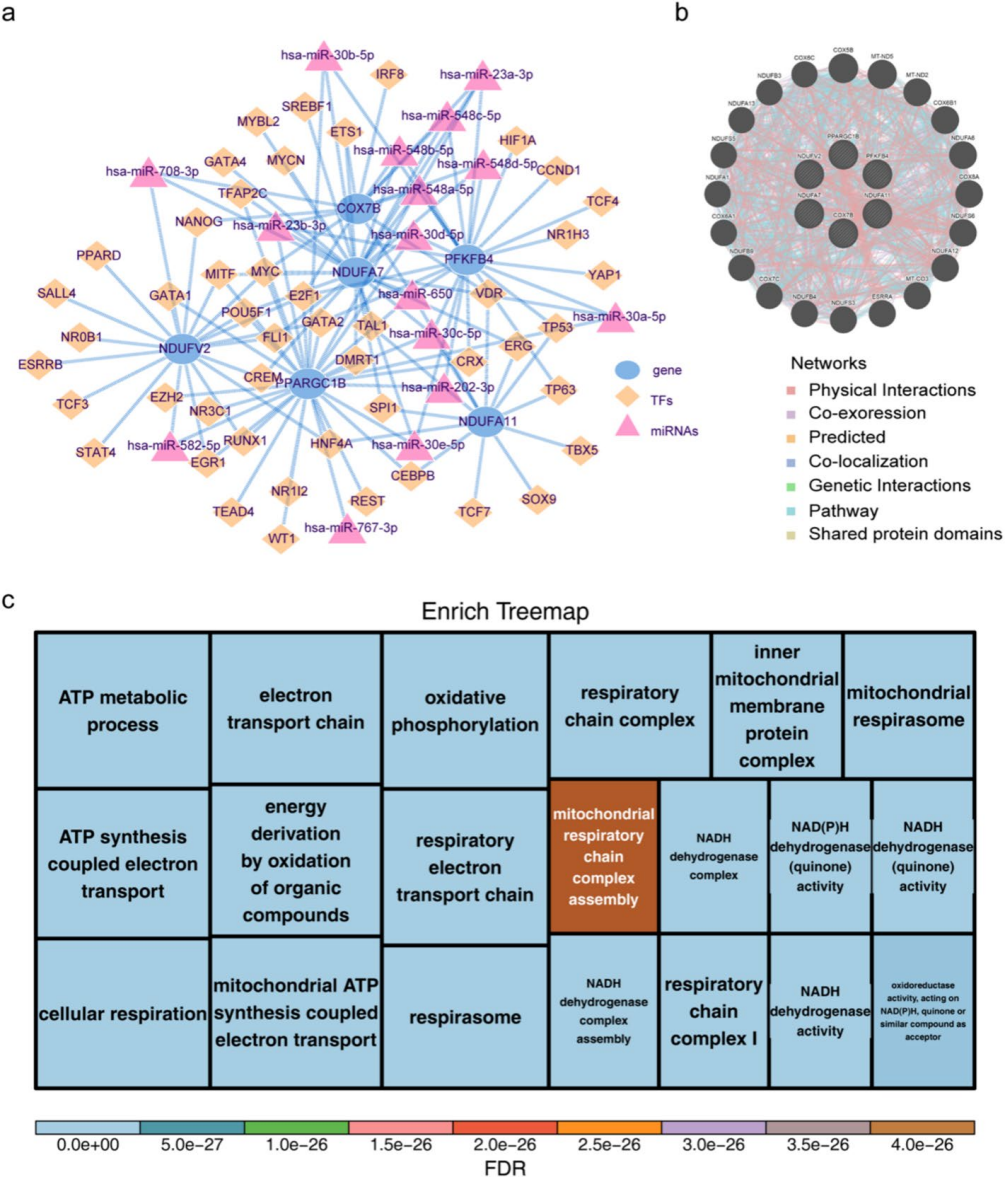
TF-miRNA-mRNA regulatory and co-expression networks of biomarkers. **a **TF-miRNA-gene regulatory network of prognostic biomarkers. **b** Co-expression network of biomarkers and GeneMANIA model genes. **c** Functional treemap of the co-expression network

To further investigate the regulatory mechanisms of biomarkers, TF-miRNA-mRNA and co-expression regulatory networks were constructed. In total, 16 miRNAs and 48 TFs that corresponded to biomarkers were identified. Following this, a TF-miRNA-mRNA regulatory network with 70 nodes (six mRNAs: COX7B, PPARGC1B, NDUFA11, PFKFB4, NDUFV2 and NDUFA7), 16 miRNAs (hsa-miR-708-3p, hsa-miR-30e-5p, etc.) and 48 TFs (microphthalmia-associated TF (MITF), CREM, repressor element-1-silencing transcription (REST), vitamin D receptor (VDR), etc.) with 128 edges was created (Fig. 9a). Specific miRNA-mRNA pairs included hsa-miR-582-5p-NDUFV2, etc., and miRNA-TFs pairs contained hsa-miR-23a-3p-PFKFB4, etc. The top 20 genes (mitochondrially encoded NADH: ubiquinone oxidoreductase core subunit 2 (MT-ND2), COX6B1, NDUFA6, COX8A, etc.) that may interact with prognostic biomarkers are depicted in Fig. 9b. Their interactions are primarily intended to regulate ATP metabolism, NADH dehydrogenase activity, mitochondrial respiratory chain complex assembly and other pathways (Fig. 9c).

### Annotation of 13 cell populations in the ccRCC-associated scRNA-seq dataset

**Fig. 10:**
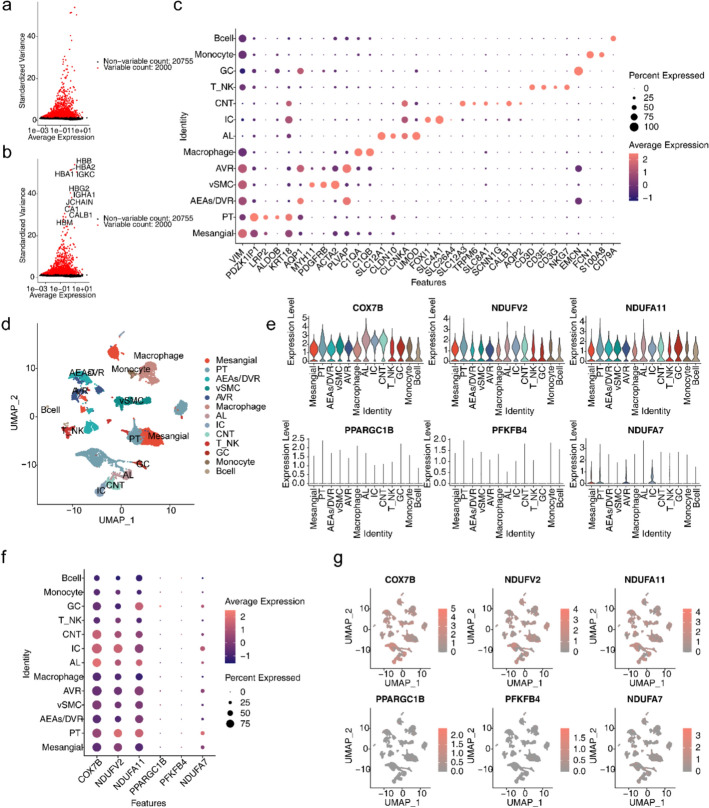
Expression analysis of biomarkers in different cell subtypes. **a**, **b** Genes with highly variable intercellular expression. **c** Expression of marker genes across different cell populations. **d** Distribution of umap cell clustering by cell subpopulation maps. **e** Violin plots for the expression analysis of six biomarkers. **f** Expression of six biomarkers across different cell populations. **g** Distribution of six biomarkers in cell clustering groups

**Fig. 11:**
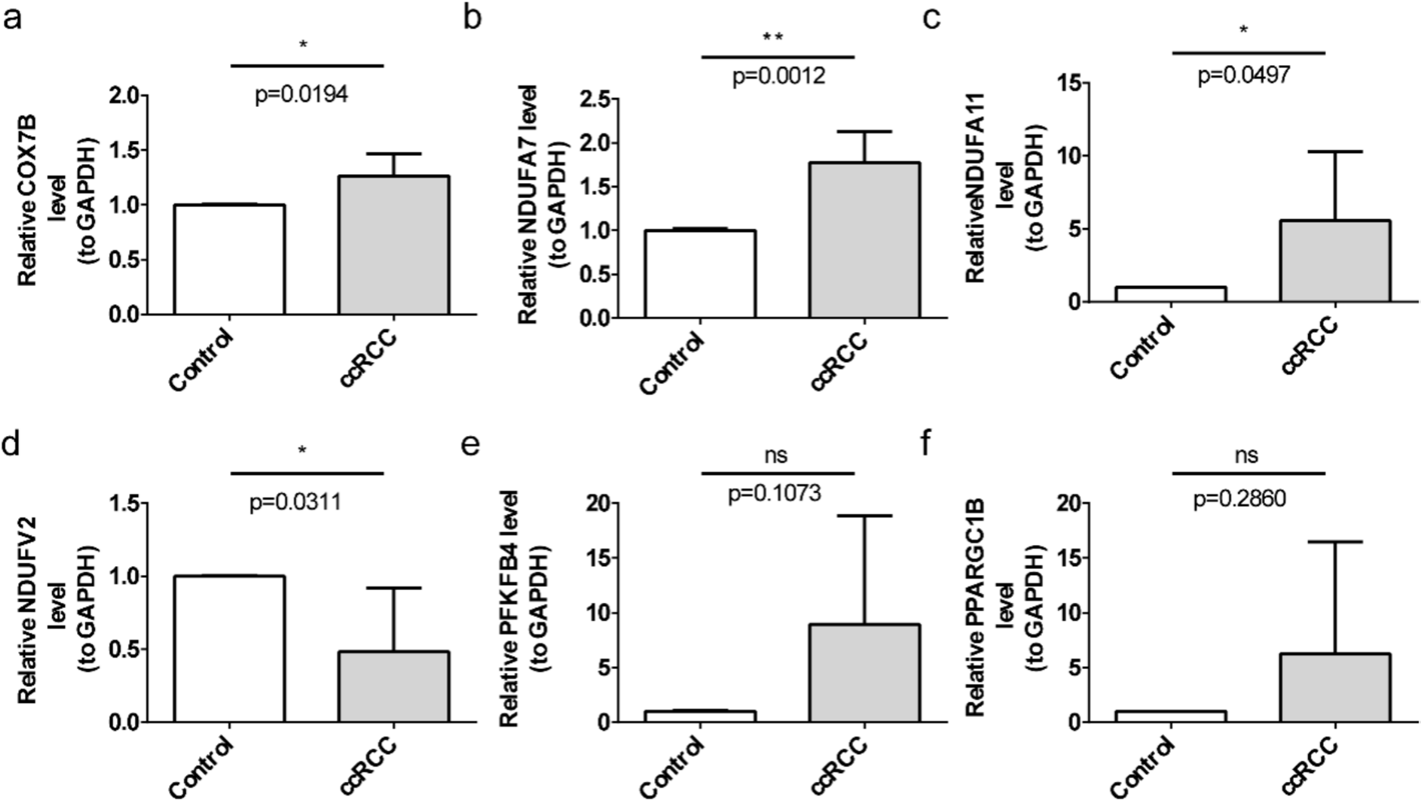
Results of qRT-PCR analysis of differential genes. **a** Bar graph of the qRT-PCR results for COX7B. **b** Bar graph of the qRT-PCR results for NDUFA7. **c** Bar graph of the qRT-PCR results for NDUFA11. **d** Bar graph of the qRT-PCR assay results for NDUFV2. **e** Bar graph of the qRT-PCR assay results for PFKFB4. **f **Bar graph of the qRT-PCR assay results for PPARGC1B. ns: no significance; *p < 0.05; **p < 0.01

Following pre-processing and standardisation, a total of 22,755 genes from 16,774 cells were obtained for subsequent analysis (Fig. S2a). Cells were filtered and standard data were processed to obtain 2,000 highly variant genes (Fig. 10a, b). The PCA was performed, and the first 30 principal components of inflection points were chosen for subsequent analysis (Fig. S2b). As a result, unsupervised clustering categorized these cells into 34 clusters, which were visualized by use of UMAP (Fig. S2c, Table S1). Based on the marker gene expression patterns in every cluster, these clusters could be divided into 13 cell subtypes (Mesangial, PT, AEAs/DVR, vSMC, etc.) (Fig. 10c, d, Fig. S3). The expression analysis showed that the expression of COX7B, NDUFA11 and NDUFV2 was higher in each cell subtype, and the biomarkers exhibited higher expression levels in renal tubular epithelial cells (PT, AL, IT, CNT, and GC) (Fig. 10e–g). The findings of qRT-PCR indicated that COX7B, NDUFA11 and NDUFA7 were enhanced in the ccRCC group, whereas NDUFV2 was decreased (Fig. 11a–f).

**Fig. 12:**
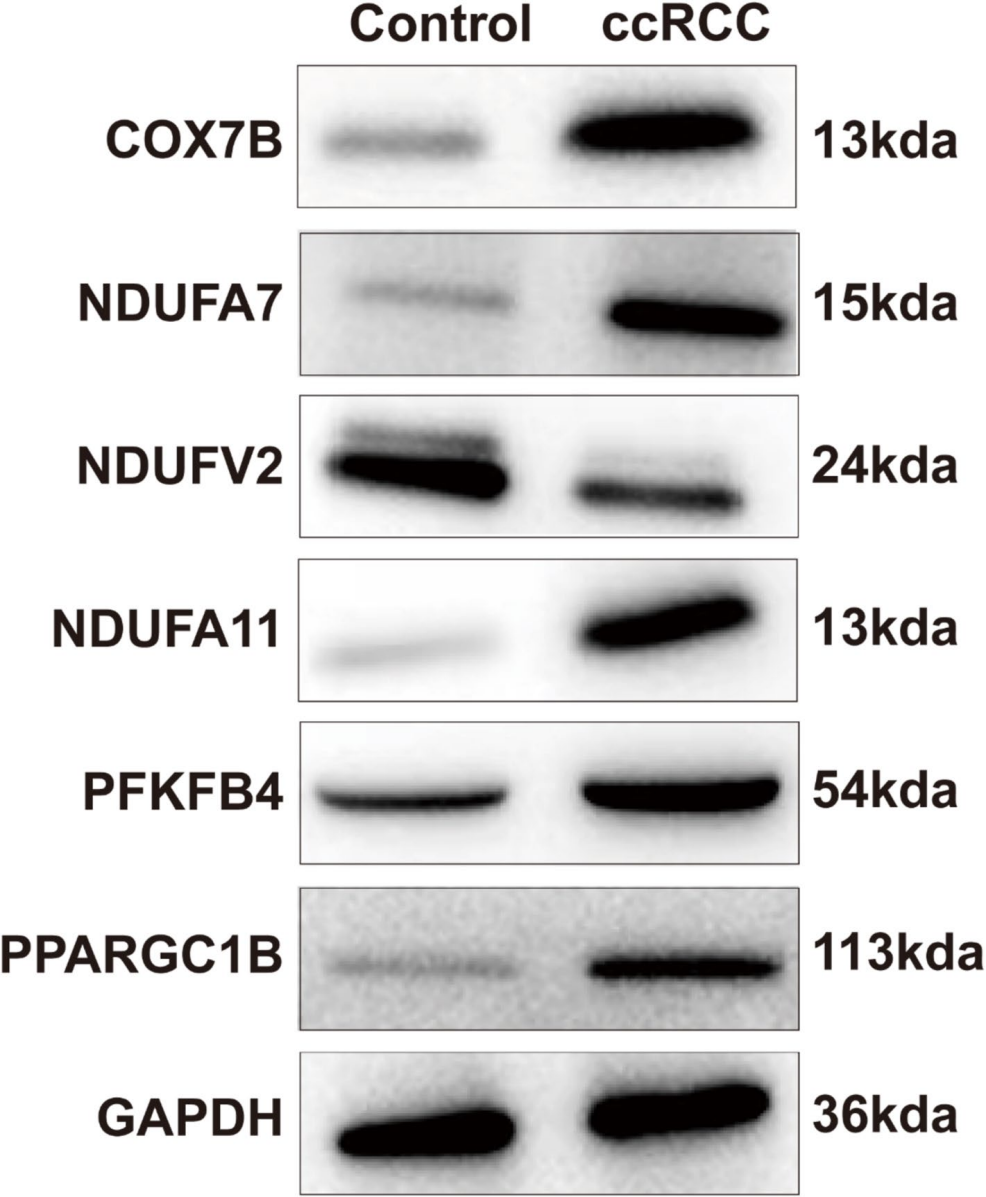
Results of WB analysis of biomarkers

Based on the qRT-PCR experiments, an additional step was undertaken to substantiate the reliability of experimental outcomes. Western blot (WB) analysis was employed to assess the protein expression profiles across two distinct tissue types. The findings of this study mirrored those of the qPCR, which revealed that COX7B, NDUFA7, NDUFA11, PFKFB4 and PPARGC1B were up-regulated in ccRCC patients when juxtaposed with the control group. Conversely, a down-regulation of NDUFV2 was observed (Fig. 12). These corroborating results serve to reinforce the validity of initial observations.

## Discussion

For ccRCC, it is the most common and well-studied RCC subtype. Its high recurrence and metastasis rates have a great impact on patients' health and quality of life [26]. The primary discovery of this study is the identification of a novel risk model based on six mitochondrial energy metabolism-related biomarkers—COX7B, PPARGC1B, NDUFA11, PFKFB4, NDUFV2 and NDUFA7. With its predictive accuracy reflected in metrics such as AUC, specificity and sensitivity, this model provides a robust tool for patient prognosis. Functional enrichment analysis highlighted that these biomarkers are enriched in critical BPs and signaling pathways, like fatty acid metabolism and PPAR signaling, which are integral to the metabolic reprogramming of ccRCC. These findings not only support the understanding of the pathogenesis of ccRCC but also suggest potential targets for therapeutic intervention. Immune cell correlation analysis revealed significant associations between biomarkers and various immune cell types in the tumor microenvironment, indicating a complex interplay between mitochondrial metabolism and immune responses that may influence immune evasion and infiltration.This relationship underscores that it is important to take into account the immune contexture in developing ccRCC treatment strategies. Drug sensitivity analysis identified specific chemotherapeutic agents exhibiting altered sensitivity based on the expression of the biomarkers in this study. These findings could guide clinicians in making more informed decisions regarding drug administration, which potentially improves treatment efficacy and reduces adverse effects. The qRT-PCR results provided a critical validation of the gene expression patterns initially observed through scRNA-seq, which demonstrated a significant concordance between the two methods. The selected genes—COX7B, PPARGC1B, NDUFA11, PFKFB4, NDUFV2 and NDUFA7—exhibited consistent expression levels, which reinforced the reliability of the biomarkers identified. Additionally, the results from WB further corroborated the findings from qRT-PCR and scRNA-seq.

The metabolic reprogramming of ccRCC involves not only enhanced glycolysis but also its partitioning—a process where glycolytic intermediates are diverted toward biosynthetic pathways (e.g., pentose phosphate pathway for nucleotide synthesis) rather than complete oxidation [4, 47, 66]. This rerouting supports rapid biomass accumulation in proliferating tumor cells. Our identification of PFKFB4 (a key regulator of glycolytic flux) as a protective biomarker aligns with this mechanism, as its overexpression may compensate for impaired mitochondrial energy production by optimizing carbon allocation.

Central to ccRCC pathogenesis is the suppression of mitochondrial oxidative phosphorylation (OxPhos), driven by VHL-HIF axis activation and mutations in electron transport chain (ETC) genes [5, 52]. This impairment forces cancer cells to rely on reductive glutamine metabolism and glycolysis for ATP generation[4]. Our risk model genes (NDUFA7, NDUFA11, NDUFV2, COX7B) encode ETC subunits, and their dysregulation (high-risk HR > 1) directly reflects compromised OxPhos capacity, correlating with poor prognosis. This reinforces mitochondria as therapeutic targets for restoring metabolic balance.

Our screening revealed 103 DE-MMRGs enriched in fatty acid metabolism and PPAR signaling—pathways pivotal to ccRCC’s metabolic identity [51, 56]. Lipid accumulation in ccRCC serves dual roles: providing energy substrates via β-oxidation and building blocks for membrane synthesis during rapid proliferation [5]. The association of PPARGC1B (a PPARγ coactivator) with favorable prognosis suggests that enhancing lipid catabolism may counteract tumor growth, whereas PFKFB4-mediated pentose phosphate flux promotes lipogenesis, driving aggressiveness [66]. In terms of genetic factors, the inactivation of the von Hippel-Lindau (VHL) gene is the most common genetic alteration in ccRCC. This leads to the increased stabilization of hypoxia-inducible factors (HIFs), which promote glycolysis and inhibit mitochondrial oxidative phosphorylation. This also results in the increased expression of certain fatty acid metabolizing enzymes, which thereby promotes lipid accumulation and tumor growth [25, 35]. Meanwhile, in terms of signaling pathways, the PPAR signaling pathway is crucial in fatty acid metabolism. Activating PPAR gamma (PPARγ) could promote the storage of fatty acids and the formation of lipid droplets, which contributes to energy storage and biofilm synthesis in tumor cells, and thus promotes or inhibits tumor growth [22, 57]. In addition, DNA methylation and histone modification alter the expression levels of metabolic enzyme genes, which affects the synthesis, metabolism and transport of fatty acids [16, 59]. These findings reveal the complex regulatory network of fatty acid metabolism in ccRCC and how it is linked to the metabolic status, proliferative capacity and pathological characteristics of tumors.

ccRCC is distinguished by dense immune infiltration, where immunosuppressive cells dominate the TME. Tumor-associated macrophages (TAMs) polarized to M2 phenotypes, regulatory T cells (Tregs), and MDSCs collectively establish an immunosuppressive milieu by secreting IL-10, TGF-β, and other cytokines that inhibit cytotoxic T cell function [75, 77]. Our data corroborate this: high-risk patients showed elevated Tregs and M1 macrophages, which paradoxically may promote inflammation without antitumor efficacy. These infiltrates correlate with poor prognosis and primary resistance to ICIs (e.g., anti-PD-1) by disrupting immune synapse formation and promoting T cell exhaustion [39, 49].

Mitochondrial metabolic rewiring directly modulates immune cell function. First, lactate overproduction from enhanced glycolysis (driven by PFKFB4) acidifies the TME, inhibiting NK cell cytotoxicity and promoting M2 macrophage polarization [60]. Second, lipid accumulation (mediated by PPARGC1B) provides precursors for immunosuppressive eicosanoids (e.g., PGE2) and dampens dendritic cell maturation [50].Third, impaired OxPhos (reflected by dysregulated NDUFA7/NDUFA11) reduces ATP available for T cell activation while increasing adenosine—a potent immunosuppressant. Our biomarker COX7B's positive correlation with Tregs exemplifies this: dysfunctional mitochondria may enhance Treg stability through altered redox signaling [20].

Six biomarkers were observed to be associated with ccRCC. Among them, NDUFV2, PPARGC1B and PFKFB4 were regarded as prognostic protective factors for ccRCC (HR < 1), and their high expression had a positive correlation with the survival and prognosis of patients. NDUFA7, NDUFA11 and COX7B were risk factors (HR > 1) and their high expression had a negative correlation with the survival and prognosis of patients. COX7B is a component of cytochrome c oxidase (CcO), which is an important part of the mitochondrial electron transport chain. Defects in COX7B not only exert a direct impact on mitochondrial energy metabolism and function but also lead to the reduced content or altered activity of CcO, which thereby affects kidney or other organ diseases [72, 75]. COX7B is also identified as a novel biomarker for patients with esophageal cancer, and its high expression is significantly related to the poor prognosis of esophageal cancer. This suggests that esophageal cancer patients with low expression may benefit more from immunotherapy [73]. PPARGC1B is a coactivator of PPARγ. Related studies have shown that the down-regulation of PPARγ has a relationship with reduced terminal differentiation and decreased cell cycle arrest, which leads to reduced cell proliferation and tumorigenesis. Moreover, high-expression PPAGC1B shows higher sensitivity to Nilotinib and Bafetinib in cancer treatment and is clinically significant for selecting anticancer therapies [27]. Yan et al. also discovered that PPARGC1B is a novel susceptible gene for Kashin-Beck disease (KBD), which induces the occurrence and development of KBD by activating nuclear genes involved in mitochondrial respiration and biogenesis and thereby affects mitochondrial function [83]. NDUFA11, a gene related to mitochondrial function, is mainly associated with aerobic respiration in mammals, and variations in this gene are linked to severe mitochondrial complex I deficiency. NDUFA11 is also confirmed as a factor with high risk for predicting OS of patients with bladder cancer and has a negative correlation with the prognosis of patients with bladder cancer [30]. In addition, research has found that NDUFA11 is highly expressed in fibroblast clusters in single-cell RNA sequencing data, which may indicate functional differences in specific cell subtypes in ccRCC. However, specific biological effects and clinical significance require further confirmation through larger-scale studies [11]. It has been shown that limiting PFKFB4 may have a beneficial effect on cancer treatment because its ectopic expression is supportive of synthetic metabolism in p53-deficient cancer cells [90]. PFKFB4 is associated with resistance to sunitinib drugs in ccRCC by mediating the pentose phosphate pathway, which affects patient treatment and prognosis to a large extent [14]. It is also related to the occurrence, progression and metastasis of gastric, breast and liver cancers, as well as glioblastoma [33, 41, 63, 80]. Wang et al. found that excessive fluoride can impede renal cell proliferation and interfere with the expression of mitochondrial complexes like NDUFV2, which leads to renal dysfunction [81]. Research also found that mitochondrial function-related genes are negatively correlated with androgen receptor (AR)-regulated genes through gene enrichment. In addition, NDUFV2 is involved in the regulation of androgens, which may be a prognostic marker and therapeutic target for prostate cancer [89]. NDUFA7 is highly expressed in the heart and its levels experience a marked decrease in a mouse model of myocardial hypertrophy. The depletion of NDUFA7 promotes the production of ROS and the activation of calmodulin protein signaling. Moreover, the depletion of NDUFA7 also contributes to cardiomyocyte hypertrophy. NDUFA7 has been confirmed to be associated with pathological cardiac hypertrophy, but research on tumor occurrence and development remains limited [71]. In summary, it was speculated that MMRGs may be involved in ccRCC occurrence and development, especially in regulating the energy supply and metabolic pathways of tumor cells.

It has been demonstrated that mitochondria are of importance to regulate cellular immunity-like innate immunity and inflammatory response [2, 84, 91]. For the past few years, immunotherapy has changed the cancer treatment paradigm, and immunologic research has once again become a focus of attention. Immunotherapy with immune-check inhibitors has been clinically effective but benefits only a minority of tumor patients. Immune infiltration in the tumor microenvironment has been observed to play an essential role in tumorigenesis and development and affects the clinical prognosis of patients with cancer. This study delved into the complicated relationship of mitochondrial function with the immune landscape in ccRCC and uncovered a complex interplay that could redefine the proposed approach to immunotherapy. Recognized for their role in cellular immunity and inflammatory responses, mitochondria have emerged as key regulators of the tumor microenvironment. The findings of this study reveal that specific immune cell populations such as activated NK cells, Tregs and CD4 memory-resting T cells in addition to M1-type macrophages are present in higher ratios in high-risk ccRCC patients. This association indicates a potential link between mitochondrial metabolism and immune cell function, which could have profound implications for disease prognosis and treatment response. The presence of Tregs in ccRCC tumors is particularly noteworthy, as they are known to suppress antitumor immune responses, which potentially gives rise to poor prognosis [43]. In this study, it was shown that Tregs and NK cells are significantly positively correlated with the expression of mitochondrial respiratory chain components such as COX7B, NDUFA11 and NDUFA7. This correlation underscores the importance of mitochondrial metabolism in regulating the suppression function of Tregs and the efficacy of NK cells in immune surveillance. As indicated by the high expression of these biomarkers, enhanced mitochondrial function may directly affect energy production and indirectly regulate the generation of regulatory signaling molecules, which thereby influences immune responses. This relationship could be pivotal in the development of new immunotherapies that target mitochondrial function to modulate the activity of immune cells.

The combination of immune checkpoint and tyrosine kinase inhibitors (ICIs and TKIs) is a hot regimen in current studies on RCC treatment. Pabolizumab-abcitinib and avelumab-abcitinib are used for the first-line treatment of metastatic RCC [8, 65]. Nevertheless, how to screen the population for maximum benefit and least adverse effects of combination therapy remains an urgent challenge. In the prognostic model established in this study, the low-risk group was more effective in immunotherapy and more sensitive to various tyrosine kinase inhibitors such as PF-562271, SB-216763, VX-702 and PLX4720 compared to the high-risk one. It can be hypothesized that this type of patient may be a beneficiary population for the combination of immuno-targeted therapy. Critically, the TME composition heavily dictates therapeutic responses. High-risk patients (enriched with Tregs/MDSCs) show primary resistance to ICIs due to: (1) upregulated checkpoint molecules (e.g., TIM-3, LAG-3) beyond PD-1 [37]; (2) exclusion of CD8 + T cells from tumor nests [49] Conversely, low-risk patients—with favorable immune infiltration (e.g., cytotoxic CD8 + T cells)—respond better to PD-1 blockade [39]. Our drug sensitivity data align with this: low-risk patients exhibited higher sensitivity to TKIs (e.g., sunitinib) partly because vascular normalization in "immune-hot" TMEs improves drug delivery [20]. Thus, our risk model stratifies patients for precision immunotherapy-tyrosine kinase inhibitor (ICI-TKI) combinations. In the present study, specific drugs and their relevance to ccRCC were explored. Drugs like sunitinib, a tyrosine kinase inhibitor, are used in ccRCC treatment due to their ability to inhibit tumor angiogenesis. However, drug resistance remains a challenge, and this study aimed to identify biomarkers predicting therapeutic response. Newer drugs targeting metabolic pathways altered in ccRCC, like metformin known for its anticancer effects and impact on mitochondrial function, were also explored. Promising drugs in preclinical models, including PFKFB4 inhibitors targeting the pentose phosphate pathway, were discussed, which could enhance the efficacy of existing therapies. The role of immunomodulatory drugs enhancing the function of cytotoxic T or NK cells was discussed as well. In conclusion, the drugs identified in this study offer opportunities for the treatment of ccRCC. Understanding their functions and potential synergies with the discovered biomarkers allows for the better tailoring of treatment strategies.

## Conclusion

In summary, six novel biomarkers linked to mitochondrial energy metabolism in ccRCC were introduced in this research and showed higher expression in stromal cells. This study identified that novel biomarkers are associated with mitochondrial energy metabolism, and demonstrate higher sensitivity and specificity in early diagnosis and predictive accuracy for treatment response compared with traditional markers. These biomarkers offer insights into the mitochondrial function and immune microenvironment of tumors, and aid in precise patient stratification and tailored treatment strategies. Moreover, in this research, an innovative patient management approach in the field of ccRCC was introduced, and a risk assessment model was developed based on key biomarkers. This model assists physicians in accurately identifying high-risk patients requiring close monitoring and facilitates the creation of personalized treatment plans, where risk scores guide therapy selection and dosage. However, this research also has certain limitations. For example, the datasets used in this research were derived from GEO and TCGA databases, which provided a rich informational foundation for analysis. However, biases within the datasets, together with the small clinical validation sample size, may limit the generalizability of the study’s conclusions. Issues such as data missingness, selection bias and potential measurement errors that could affect the accurate estimation of variable relationships, and confounding factors that may obscure clear causal interpretations were also noted. Future research on ccRCC should be aimed at expanding sample sizes, reducing biases, controlling for confounders, and employing rigorous methodologies to deepen understanding. The key focus should be on validating biomarkers through functional experiments to make sure that the findings are reliable and practical. Moreover, integrating multi-omics data will be crucial to revealing a comprehensive, multidimensional picture of disease mechanisms. Future research will further explore the roles played by these genes in the tumor microenvironment and immune functions, and integrate these biomarkers into clinical practice to advance ccRCC treatment and personalized medicine.

## Supplementary Information

Supplementary material 1.

## Data Availability

The datasets generated and/or analyzed during the current study are available in the Gene Expression Omnibus (GEO) repository under accession numbers GSE159115 (https://www.ncbi.nlm.nih.gov/geo/query/acc.cgi?acc=GSE159115) and GSE29609 (https://www.ncbi.nlm.nih.gov/geo/query/acc.cgi?acc=GSE29609). Additionally, the transcriptomic and clinical data for kidney renal clear cell carcinoma (KIRC) were obtained from The Cancer Genome Atlas (TCGA) database via the GDC portal (https://portal.gdc.cancer.gov/). These datasets were accessed on July 13, 2023.
