# Supplementary material for: Mitochondrial energy metabolism genes as prognostic biomarkers in clear cell renal cell carcinoma via single-cell and bulk RNA sequencing analyses
Source: Discov Oncol. 2025 Dec 9;17:77. doi: 10.1007/s12672-025-04224-1 (PMC12799841; doi:10.1007/s12672-025-04224-1)
Supplement: Supplementary file 1 — Supplementary material 1. [file 12672_2025_4224_MOESM1_ESM.docx]

## Supplementary Figure


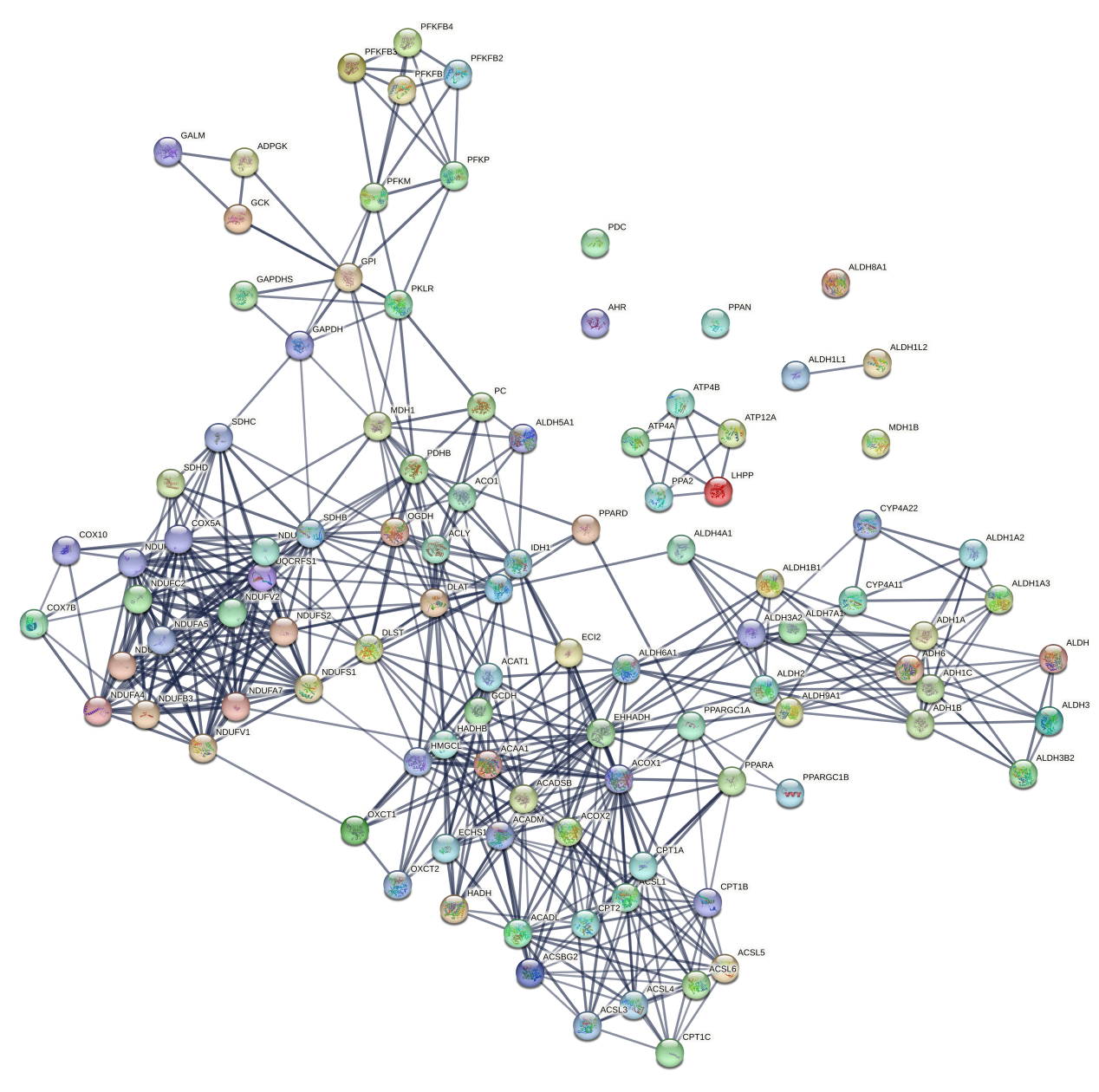


**Fig.S1** Protein-protein interaction (PPI) network for DE-MMRGs.


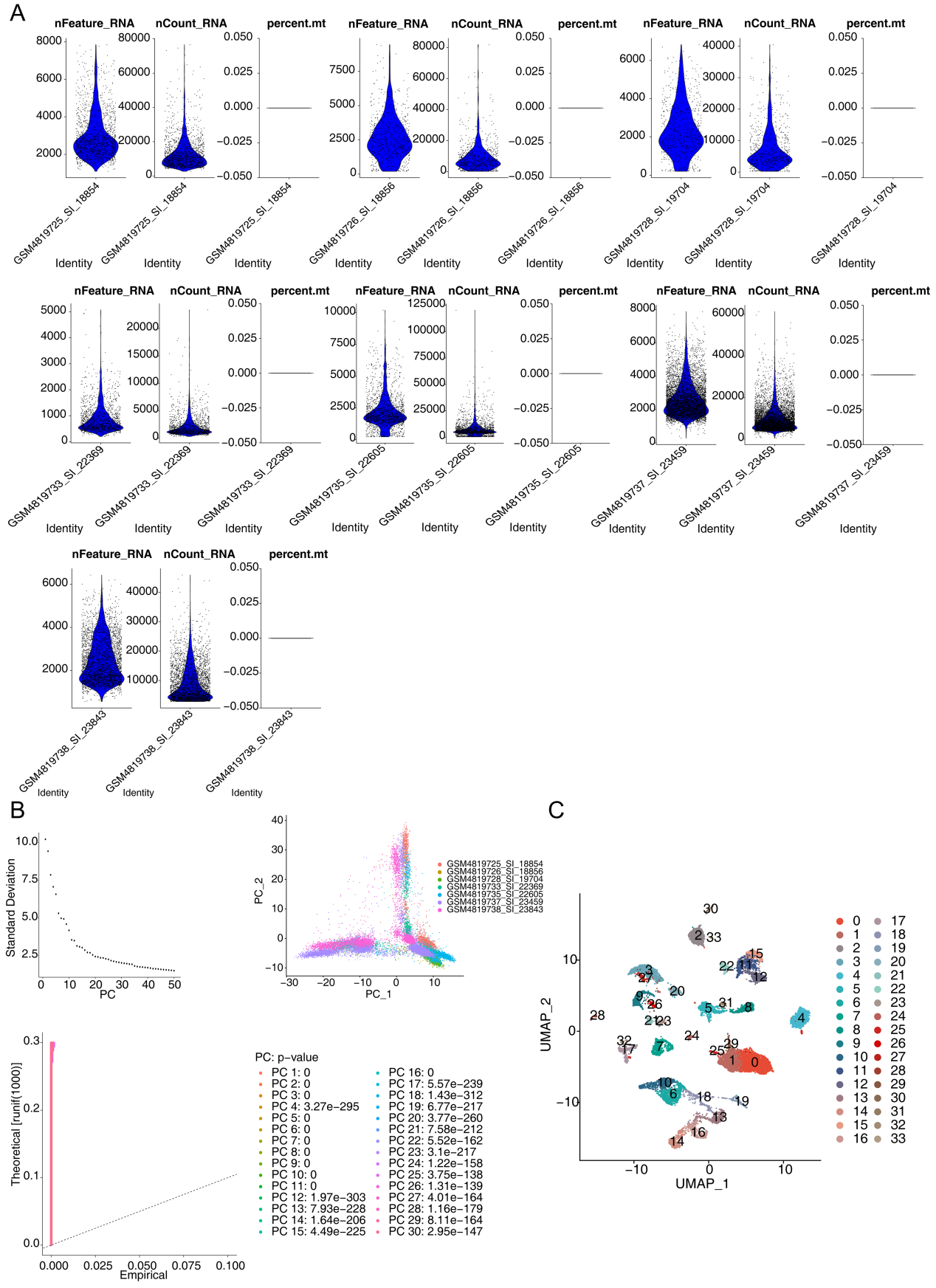


**Fig.S2** The scRNA-seq dataset analysis.(A) The scRNA-seq data filtering. (B) Examine and visualize PCA results with ElbowPlot. (C) Cell clustering visualization.


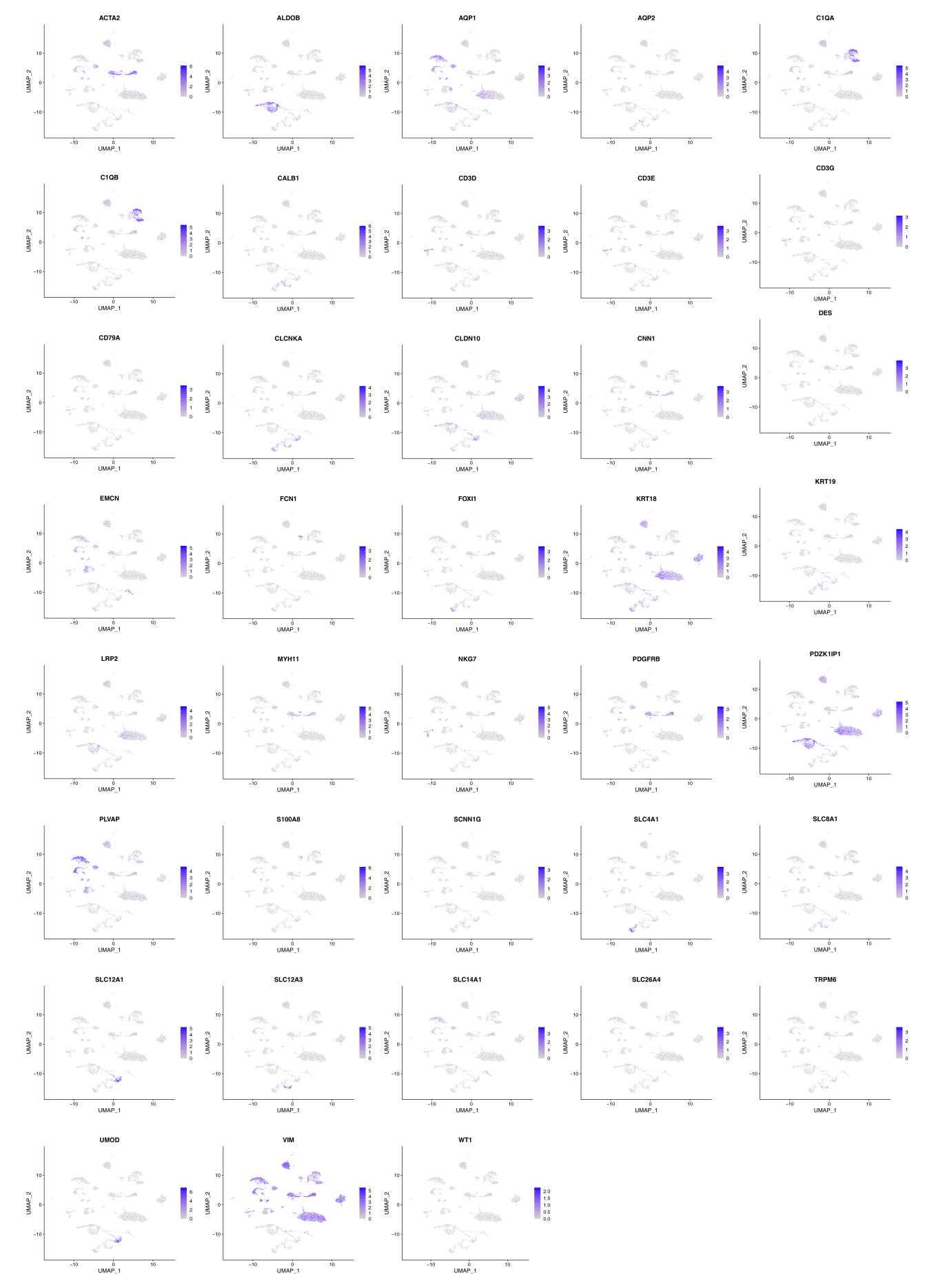


**Fig.S3** Scatterplot of marker gene expression across distinct cell populations.

**Table.S1** Number of cells per cluster.

|  | 0 | 1 | 2 | 3 | 4 | 5 | 6 | 7 | 8 | 9 | 10 | 11 | 12 | 13 | 14 | 15 | 16 |
| --- | --- | --- | --- | --- | --- | --- | --- | --- | --- | --- | --- | --- | --- | --- | --- | --- | --- |
| Cell count | 2137 | 1291 | 1192 | 1022 | 1001 | 838 | 787 | 664 | 658 | 645 | 604 | 590 | 533 | 525 | 507 | 487 | 430 |
|  | 17 | 18 | 19 | 20 | 21 | 22 | 23 | 24 | 25 | 26 | 27 | 28 | 29 | 30 | 31 | 32 | 33 |
| Cell count | 419 | 357 | 289 | 282 | 228 | 211 | 209 | 136 | 105 | 105 | 104 | 93 | 87 | 81 | 80 | 55 | 22 |
